# Maternal and perinatal factors are associated with risk of pediatric central nervous system tumors and poorer survival after diagnosis

**DOI:** 10.1038/s41598-021-88385-3

**Published:** 2021-05-17

**Authors:** Maral Adel Fahmideh, Erin C. Peckham-Gregory, Jeremy M. Schraw, Murali Chintagumpala, Stephen C. Mack, Philip J. Lupo, Michael E. Scheurer

**Affiliations:** 1 Department of Medicine, Section of Epidemiology and Population Sciences, Baylor College of Medicine, Houston, TX USA; 2 Dan L. Duncan Comprehensive Cancer Center, Baylor College of Medicine, Houston, TX USA; 3 Department of Pediatrics, Center for Epidemiology and Population Health, Baylor College of Medicine, Houston, TX USA; 4 Section of Hematology-Oncology, Department of Pediatrics, Baylor College of Medicine, Houston, TX USA; 5 Texas Children’s Cancer Center, Texas Children’s Hospital, Houston, TX USA

**Keywords:** Medical research, Oncology, Risk factors

## Abstract

Central nervous system (CNS) tumors are the most common solid tumors in children. Findings on the role of maternal and perinatal factors on the susceptibility or outcome of these tumors are inconclusive. Therefore, we investigated the association between these early-life factors, risk, and survival of pediatric CNS tumors, using data from one of the world’s largest and most diverse cancer registries. Information on pediatric CNS tumor cases (*n* = 1950) for the period 1995–2011 was obtained from the Texas Cancer Registry. Birth certificate controls were frequency-matched on birth year at a ratio of 10:1 for the same period. Evaluated maternal and perinatal variables were obtained from birth records. Unconditional logistic regression was used to generate adjusted odds ratios (ORs) and 95% confidence intervals (CIs) for etiological factors. Additionally, Cox proportional hazards regression was employed to assess adjusted hazard ratios (HRs) and 95% CIs for survival factors. The results indicated that Hispanic and non-Hispanic black mothers were less likely to have children with CNS tumors compared to non-Hispanic white mothers (OR 0.88 [95% CI 0.78–0.98] *P*-value = 0.019; OR 0.79 [95% CI 0.67–0.93 *P*-value = 0.004], respectively). Infants born large for gestational age (OR 1.26 [95% CI 1.07–1.47] *P*-value = 0.004) and those delivered pre-term (OR 1.19 [95% CI 1.04–1.38] *P*-value = 0.013) showed an increased risk of CNS tumors. Infants born by vaginal forceps or vacuum delivery had a higher risk of CNS tumors compared to those born by spontaneous vaginal delivery (OR 1.35 [95% CI 1.12–1.62] *P*-value = 0.002). Additionally, offspring of Hispanic and non-Hispanic black mothers showed a higher risk of death (HR 1.45 [95% CI 1.16–1.80] *P*-value = 0.001; HR 1.53 [95% CI 1.12–2.09] *P*-value = 0.008, respectively). Infants born by cesarean had a higher risk of death compared to those delivered vaginally (HR 1.28 [95% CI 1.05–1.57] *P*-value = 0.016). These findings indicate the important role of maternal and perinatal characteristics in the etiology and survival of these clinically significant malignancies.

## Introduction

Primary central nervous system (CNS) tumors are the most common solid tumors in children and remain a significant contributor to death by disease in this population^1^. These tumors are heterogeneous and consist of neoplasms arising from the brain, cranial nerves, spinal nerves, and meninges; of which brain tumors occur most commonly in children^1^. The incidence of CNS tumors in children and adolescents ≤ 19 years of age in United States (U.S.) from 2012 to 2016 was 6.06 per 100,000 children^2^. The ten-year survival rate for all pediatric CNS tumors was estimated at 68.7% with lowest (45.6%) and highest (97.8%) survival rates being attributable to brain stem tumors and tumors of the cranial nerves, respectively^1^. Despite their clinical significance, the factors contributing to the pathogenesis, etiology, and prognosis of pediatric CNS tumors remain largely undetermined.

The only established risk factors for pediatric brain tumors are exposure to moderate-to-high doses of ionizing radiation and some rare genetic syndromes. There is also evidence of positive associations between pediatric brain tumor risk and birth defects, markers of fatal growth, advanced parental age, maternal dietary N-nitroso compounds (NOCs), and exposure to pesticides^3–5^. However, due to the heterogeneity of these malignancies and their likely diverse etiologies, further investigations to identify specific risk factors for each histological subtype are required^4,5^. Further, the primary factors predicting survival for pediatric CNS tumors are currently tumor histology, location, and patient age^6^; although molecular prognostic markers have recently been identified for some histological subtypes and are used for prognostic stratification^7,8^. While there is evidence that pediatric CNS tumors arise in early-life, knowledge on the role of maternal and perinatal factors on susceptibility or outcome of these malignancies is limited.

Given a general lack of confirmed risk and prognostic factors for pediatric CNS tumors, we sought to identify the role of perinatal and maternal factors on the risk and outcome of pediatric CNS tumors using data from the Texas Cancer Registry (TCR), one of the world’s largest and most diverse cancer registries.

## Methods

### Study design and setting

We conducted a case–control study to investigate the association between perinatal and maternal characteristics and risk of CNS tumors in children. Additionally, a cohort design was employed to examine the effect of perinatal and maternal factors on the survival of patients with pediatric CNS tumors. Data from the TCR and the Texas Center for Health Statistics (CHS), Department of State Health Services, were used to identify the eligible population. The eligible population included individuals who were born in Texas during the period between 1 January 1995 and 31 December 2011. The TCR was “Gold Certified” by the North American Association of Central Cancer Registries during the study period^9^. Informed consent was not required for this study since it was registry based and deidentified and anonymous data were available for analyses. All methods were carried out in accordance with relevant guidelines and regulations. The study was approved by the Institutional Review Boards at Baylor College of Medicine and the Texas Department of State Health Services.

### Participant ascertainment

Eligible cases were defined as children, up to 16 years old, born and diagnosed with a primary CNS tumor in Texas during the study period. Cases were identified through the TCR as those with a group III code according to the International Classification of Childhood Cancer, third edition (ICCC-3)^10^, which is based on the International Classification of Diseases for Oncology, third edition (ICD-O-3)^11^. Cases of astrocytoma, medulloblastoma and ependymoma, the most common histological subtypes of brain tumors, were defined according to ICCC-3, group III (ICD-O-3 morphology codes provided) as following: ependymoma—IIIa (9383, 9391–9394); astrocytoma—IIIb (9380, 9384, 9400–9411, 9420, 9421–9424, 9440–9442); and medulloblastoma—IIIc (9470–9472, 9474, 9480).

For the case–control analysis, eligible controls were randomly selected among the birth certificate records of children who were born in Texas during the same period as cases (1995–2011) and were confirmed not to be included in the TCR. Controls were frequency matched to cases on birth year at a ratio of ten control subjects for every case with a CNS tumor.

### Perinatal and maternal variables

Perinatal and maternal characteristics for cases and controls were identified through Texas birth certificates provided by the CHS. The following variables were included in the analysis, either as predictors for risk and outcome or potential confounders: infant sex (male vs. female); birth year (continuous variable); plurality (singleton vs. higher order); birth order based on the history of other live births (1, 2, and ≥ 3); gestational age at birth (< 37, 37–41, ≥ 42 weeks); birth weight (< 2500, 2500–3999, ≥ 4000 g [g]); size for gestational age (small [< 10th percentile], appropriate, large [> 90th percentile]); mode of delivery (vaginal spontaneous, vaginal forceps or vacuum, and cesarean); maternal race/ethnicity (non-Hispanic white, non-Hispanic black, Hispanic, other); maternal age at delivery (< 25, 25–29, 30–34, ≥ 35 years); maternal education (< high school, high school, > high school); maternal nativity (U.S., Mexico, other foreign country); maternal residence on Texas-Mexico border at the time of delivery (yes vs. no); maternal residency at delivery in urban vs. rural areas; maternal smoking during pregnancy (yes vs. no); and pre-pregnancy maternal body mass index (BMI) (≤ 18.5, 18.5–25, 25–29, ≥ 30 kg/m^2^). Maternal BMI was calculated from 2005 onward since maternal height and pre-pregnancy weight were not recorded on Texas birth certificates prior to 2005.

### Statistical analysis

#### Case–control analysis: etiology

Unconditional logistic regression was used to estimate the crude association between each maternal or perinatal factor and the risk of pediatric CNS tumors overall, as well as the most common histological subtypes (astrocytoma, ependymoma, and medulloblastoma). The results of logistic regression models were later adjusted for infant sex, birth year, maternal education and maternal race/ethnicity, based on a priori knowledge.

#### Cohort analysis: survival

Cox regression was employed to assess the effect of maternal or perinatal factors on survival for all pediatric CNS tumors combined and the most common histological subtypes individually. The results of Cox regression models were later adjusted for infant sex, birth year, maternal education, maternal race/ethnicity, and tumor malignancy, based on a priori knowledge. Survival time was defined as the date of diagnosis to the date of death or the end of the study follow-up; 31 December 2012. Kaplan–Meier plots were used to show the overall survival in patients in relation to investigated perinatal and maternal factors.

Because of the number of statistical tests performed, we also provide the reference *P*-value for an experiment-wide significance with the Bonferroni’ correction.

The analyses were conducted using Stata statistical software version 16 (StataMP, College Station, TX, USA).

## Results

In total, we identified 1950 cases with pediatric primary CNS tumors, and 19,500 controls. Distribution of the most common histological subtypes was as follows: 978 astrocytoma, 218 medulloblastoma, and 157 ependymoma. The demographic characteristics of cases are summarized in Table 1 and the distributions of maternal and perinatal characteristics among cases and controls are summarized in Tables 2 and 3 as well as Supplementary Tables 1–4.

**Table 1:** Demographic characteristics of children diagnosed with central nervous system tumors.

Demographic characteristics	All CNS cases	Astrocytoma	Ependymoma	Medulloblastoma
Number	1934	970	157	217
**Infant sex, n (%)**				
Male	1032 (53.4)	496 (51.1)	90 (57.3)	143 (65.9)
Female	902 (46.6)	474 (48.9)	67 (42.7)	74 (34.1)
**Maternal race/ethnicity, n (%)**				
Non-hispanic white	874 (45.2)	480 (49.5)	61 (38.8)	98 (45.2)
Non-hispanic black	194 (10.0)	101 (10.4)	10 (6.4)	17 (7.8)
Hispanic	807 (41.7)	357 (36.8)	82 (52.2)	96 (44.2)
Other	59 (3.1)	32 (3.3)	4 (2.6)	6 (2.8)
**Birth year, n (%)**				
1995–1999	810 (41.88)	406 (41.86)	62 (39.49)	93 (42.86)
2000–2004	688 (35.57)	358 (36.91)	56 (35.67)	83 (38.25)
2005–2011	436 (22.54)	206 (21.24)	39 (24.84)	41 (18.89)
**Diagnosis year, n (%)**				
1995–1999	117 (6.05)	55 (5.67)	19 (12.10)	14 (6.45)
2000–2004	422 (21.82)	231 (23.81)	35 (22.29)	54 (24.88)
2005–2011	1395 (72.13)	684 (70.52)	103 (65.61)	149 (68.66)
**Diagnosis age, n (%)**				
< 1	214 (11.07)	79 (8.14)	20 (12.74)	12 (5.53)
1–5	963 (49.79)	520 (53.61)	91(57.96)	118 (54.38)
6–10	536 (27.71)	288 (29.69)	31 (19.75)	67 (30.88)
> 10	221 (11.43)	83 (8.56)	15 (9.55)	20 (9.22)

**Table 2:** Significant associations between maternal and perinatal factors and risk of pediatric central nervous system tumors.

Maternal and perinatal characteristics	All nervous system tumors							
	Cases	Controls	Unadjusted model			Adjusted model^a^		
			OR	95% CI	P-value^b^	OR	95% CI	P-value^b^
**Maternal race/ethnicity**								
Non-hispanic white	874 (45.2)	7942 (41.0)	Reference			Reference		
Non-hispanic black	194 (10.0)	2254 (11.7)	0.78	0.66–0.92	0.003	0.79	0.67–0.93	0.004
Hispanic	807 (41.7)	8473 (43.8)	0.87	0.78–0.96	0.005	0.88	0.78–0.98	0.019
Other	59 (3.1)	671 (3.5)	0.79	0.61–1.05	0.110	0.76	0.57–1.01	0.060
Missing	0	0						
**Infant sex**								
Male	1032 (53.4)	9821 (50.8)	Reference			Reference		
Female	902 (46.6)	9519 (49.2)	0.90	0.82–0.99	0.031	0.89	0.81–0.99	0.024
Missing	0 (0.0)	0 (0.0)						
**Size for gestational age**								
< 10th percentile	253 (13.1)	2640 (13.7)	0.98	0.85–1.13	0.795	0.98	0.85–1.13	0.819
10th–90th percentile	1453 (75.1)	14,883 (76.9)	Reference			Reference		
> 90th percentile	200 (10.3)	1616 (8.4)	1.27	1.08–1.48	0.003	1.26	1.07–1.47	0.004
Missing	28 (1.5)	201 (1.0)						
**Gestational age**								
< 37 weeks	252 (13.0)	2203 (11.4)	1.18	1.02–1.35	0.024	1.19	1.04–1.38	0.013
37–41 weeks	1599 (82.7)	16,431 (84.9)	Reference			Reference		
≥ 42	55 (2.8)	514 (2.7)	1.09	0.83–1.46	0.511	1.12	0.84–1.48	0.444
Missing	28 (1.5)	192 (1.0)						
Continuous			0.98	0.96–1.00	0.052	0.98	0.96–0.99	0.033
**Delivery type**								
Vaginal spontaneous	1225 (63.3)	12,886 (66.6)	Reference			Reference		
Vaginal forceps or vacuum	144 (7.4)	1110 (5.7)	1.36	1.14–1.64	0.001	1.35	1.12–1.62	0.002
Cesarean	564 (29.2)	5331 (27.6)	1.11	1.00–1.24	0.045	1.10	0.99–1.23	0.067
Missing	1 (0.1)	13 (0.1)						
**Birth weight (g)**								
< 2500	166 (8.6)	1,498 (7.8)	1.14	0.96–1.35	0.131	1.15	0.97–1.37	0.105
2500–3999	1596 (82.5)	16,396 (84.7)	Reference			Reference		
≥ 4000	172 (8.9)	1433 (7.4)	1.23	1.04–1.46	0.014	1.18	0.99–1.40	0.051
Missing	0 (0.0)	13 (0.1)						
Continuous			1.00	0.99–1.00	0.207	1.00	0.99–1.00	0.541

^a^Adjusted for birth year, sex, maternal race/ethnicity, and maternal education.

^b^Bonferroni corrected reference *P* values: 0.003 for an experiment-wide significance of 0.05.

**Table 3:** Significant associations between maternal and perinatal factors and risk of pediatric astrocytoma, ependymoma, and medulloblastoma.

Maternal and perinatal characteristics	Astrocytoma							
	Cases	Controls	Unadjusted model			Adjusted model^a^		
			OR	95% CI	P-value^b^	OR	95% CI	P-value^b^
**Maternal race/ethnicity**								
Non-hispanic white	480 (49.5)	4037 (41.6)	Reference			Reference		
Non-hispanic black	101 (10.4)	1090 (11.2)	0.78	0.62–0.98	0.030	0.79	0.63–0.99	0.049
Hispanic	357 (36.8)	4225 (43.6)	0.71	0.62–0.82	0.000	0.76	0.65–0.89	0.001
Other	32 (3.3)	348 (3.6)	0.77	0.53–1.12	0.178	0.69	0.46–1.02	0.064
Missing	0 (0.0)	0 (0.0)						
**Gestational age**								
< 37 weeks	127 (13.1)	1081 (11.1)	1.21	0.99–1.47	0.059	1.24	1.02–1.52	0.029
37–41 weeks	803 (82.8)	8266 (85.3)	Reference			Reference		
≥ 42	24 (2.5)	254 (2.6)	0.97	0.64–1.49	0.898	0.99	0.66–1.54	0.979^b^
Continuous			0.98	0.95–1.01	0.163	0.98	0.95–1.00	0.098
Missing	16 (1.6)	99 (1.0)						
**Delivery type**								
Vaginal spontaneous	613 (63.2)	6512 (67.1)	Reference			Reference		
Vaginal forceps or vacuum	81 (8.4)	514 (5.3)	1.67	1.31–2.15	0.000	1.63	1.27–2.09	0.000
Cesarean	275 (28.3)	2668 (27.5)	1.09	0.94–1.27	0.233	1.09	0.94–1.27	0.266
Missing	1 (0.1)	6 (0.1)						
	**Ependymoma**							
**Maternal education**								
< High school	50 (31.9)	486 (31.0)	0.79	0.53–1.17	0.237	0.73	0.48–1.11	0.140
High school	59 (37.5)	451 (28.7)	Reference			Reference		
> High school	44 (28.0)	619 (39.4)	0.54	0.36–0.82	0.003	0.54	0.35–0.82	0.004
Missing	4 (2.6)	14 (0.9)						
**Maternal nativity**								
U.S. born	99 (63.1)	1144 (72.9)	Reference			Reference		
Mexico	40 (25.5)	268 (17.1)	1.72	1.17–2.55	0.006	1.67	1.03–2.71	0.038
Other	17 (10.8)	154 (9.8)	1.28	0.74–2.19	0.378	1.86	1.01–3.44	0.046
Missing	1 (0.6)	4 (0.2)						
**Gestational age**								
< 37 weeks	21 (13.4)	165 (10.5)	1.35	0.83–2.19	0.233	1.37	0.83–2.27	0.223
37–41 weeks	127 (80.9)	1344 (85.6)	Reference			Reference		
≥ 42	8 (5.1)	39 (2.5)	2.17	0.99–4.75	0.052	2.42	1.09–5.40	0.031
Continuous			0.97	0.91–1.04	0.436	0.98	0.92–1.05	0.599
Missing	1 (0.6)	22 (1.4)						
**Maternal BMI**^c^								
< 18.5	1 (2.6)	16 (4.1)	0.45	0.06–3.50	0.442	0.43	0.05–3.45	0.425
18.5–24.9	25 (64.1)	178 (45.6)	Reference			Reference		
25–29.9	5 (12.8)	100 (25.7)	0.36	0.13–0.96	0.041	0.33	0.12–0.91	0.033
≥ 30	8 (20.5)	94 (24.1)	0.61	0.26–1.39	0.239	0.52	0.22–1.24	0.139
Continuous			0.98	0.92–1.03	0.415	0.97	0.91–1.03	0.285
Missing	0 (0.0)	2 (0.5)						
	**Medulloblastoma**							
**Maternal nativity**								
U.S. born	166 (76.6)	1600 (73.7)	Reference			Reference		
Mexico	40 (18.4)	379 (17.5)	1.02	0.71–1.46	0.926	0.98	0.63–1.54	0.943
Other	10 (4.6)	185 (8.5)	0.52	0.27–1.00	0.051	0.42	0.18–0.96	0.040
Missing	1 (0.4)	6 (0.3)						
**Infant sex**								
Male	143 (65.9)	1095 (50.5)	Reference			Reference		
Female	74 (34.1)	1075 (49.5)	0.53	0.39–0.71	0.000	0.53	0.39–0.71	0.000
Missing	0 (0.0)	0 (0.0)						
**Birth order**								
1st	162 (74.7)	1641 (75.6)	Reference			Reference		
2nd	43 (19.7)	333 (15.3)	1.31	0.92–1.87	0.140	1.31	0.91–1.89	0.140
≥ 3rd	6 (2.8)	153 (7.1)	0.39	0.17–0.91	0.030	0.39	0.17–0.91	0.029
Continuous			0.86	0.69–1.06	0.158	0.86	0.69–1.06	0.153
Missing	6 (2.8)	43 (2.0)						

^a^Adjusted for birth year, sex, maternal race/ethnicity, and maternal education.

^b^Bonferroni corrected reference *P* values: 0.003 for an experiment-wide significance of 0.05.

^c^Pre-pregnancy maternal body mass index (BMI) data collection began in 2005.

Among the investigated factors, several were significantly associated with risk of pediatric CNS tumors overall, as well as some specific histological subtypes. As Table 2 illustrates, Hispanic and non-Hispanic black mothers were less likely to have children who developed CNS tumors compared to non-Hispanic white mothers (adjusted odds ratio (aOR) 0.88 [95% confidence interval (CI) 0.78–0.98] *P*-value = 0.019; aOR 0.79 [95% CI 0.67–0.93] *P*-value = 0.004, respectively). Compared to male children, female children were less likely to develop CNS tumors (aOR 0.89 [95% CI 0.81–0.99] *P*-value = 0.024). Conversely, infants born large for gestational age (aOR 1.26 [95% CI 1.07–1.47] *P*-value = 0.004) as well as those delivered pre-term (< 37 weeks) (aOR 1.19 [95% CI 1.04–1.38] *P*-value = 0.013) showed an increased risk of CNS tumors compared to the respective reference groups. Moreover, infants born with high birth weight (≥ 4000 g) were at higher risk of developing CNS tumors (crude OR 1.23 [95% CI 1.04–1.46] *P*-value = 0.014); however, the effect was attenuated in the adjusted model (aOR 1.18 [95% CI 0.99–1.40] *P*-value = 0.051). Compared to women with spontaneous vaginal delivery, those who delivered with vaginal forceps or experienced a vacuum delivery were more likely to have children that developed CNS tumors (aOR 1.35 [95% CI 1.12–1.62] *P*-value = 0.002). Children born by cesarean delivery were also suggested to be at increased risk (aOR 1.10 [95% CI 0.99–1.23] *P*-value = 0.067); however, this result did not reach statistical significance.

Associations between maternal and perinatal factors and risk of developing astrocytoma, ependymoma, and medulloblastoma in children are presented in Table 3. Similar to the results for all CNS tumors combined, offspring of Hispanic and non-Hispanic black mothers were less likely to develop astrocytoma compared to offspring of non-Hispanic white mothers (aOR 0.76 [95% CI 0.65–0.89] *P*-value = 0.001; aOR 0.79 [95% CI 0.63–0.99] *P*-value = 0.049, respectively). Moreover, effect estimates for pre-term birth as well as vaginal forceps or vacuum delivery were stronger for astrocytomas (aOR 1.24 [95% CI 1.02–1.52] *P*-value = 0.029; aOR 1.63 [95% CI 1.27–2.09] *P*-value = 0.000, respectively). An increased risk of ependymoma was found for offspring of native-born Mexican mothers compared to mothers born in the U.S. (aOR 1.67 [95% CI 1.03–2.71] *P*-value = 0.038). A decreased risk of ependymoma was detected for children of mothers with education greater than high school compared to those with high school education (aOR 0.54 [95% CI 0.35–0.82] *P*-value = 0.004). Females remained at a significantly lower odds of medulloblastoma compared to males (aOR 0.53 [95% CI 0.39–0.71] *P*-value = 0.000). We found potential associations between high maternal BMI, post-term birth and ependymoma as well as maternal nativity, birth order and medulloblastoma. However, these findings are based on small numbers and should be interpreted with caution (Table 3).

As Table 4 shows, several maternal and perinatal factors were associated with overall survival. Among all cases diagnosed with CNS tumors, children of Hispanic and non-Hispanic black mothers showed a higher risk of death compared to children of non-Hispanic white mothers (adjusted hazard ratio (aHR) 1.45 [95% CI 1.16–1.80] *P*-value = 0.001; aHR 1.53 [95% CI 1.12–2.09] *P*-value = 0.008, respectively). Infants born by cesarean delivery had a higher risk of death compared to those delivered vaginally (aHR 1.28 [95% CI 1.05–1.57] *P*-value = 0.016) (Table 4).

**Table 4:** Significant associations between maternal and perinatal factors and survival of pediatric central nervous system tumor cases.

Maternal and perinatal characteristics	All nervous system tumors						
	Cases	Unadjusted model			Adjusted model^a^		
		HR	95% CI	P-value^b^	HR	95% CI	P-value^b^
**Maternal race/ethnicity**							
Non-hispanic white	874 (45.2)	Reference			Reference		
Non-hispanic black	194 (10.0)	1.51	1.11–2.05	0.009	1.53	1.12–2.09	0.008
Hispanic	807 (41.7)	1.41	1.15–1.73	0.001	1.45	1.16–1.80	0.001
Other	59 (3.1)	1.69	1.05–2.76	0.033	1.71	1.04–2.82	0.036
Missing	0 (0.0)						
**Delivery type**							
Vaginal spontaneous	1225 (63.3)	Reference			Reference		
Vaginal forceps or vacuum	144 (7.4)	0.78	0.52–1.17	0.229	0.79	0.53–1.20	0.277
Cesarean	564 (29.2)	1.29	1.05–1.57	0.014	1.28	1.05–1.57	0.016
Missing	1 (0.1)						

^a^Adjusted for birth year, sex, maternal race/ethnicity, maternal education, and tumor malignancy.

^b^Bonferroni corrected reference *P* values: 0.003 for an experiment-wide significance of 0.05.

The higher risk of death among children of non-Hispanic black mothers remained significant for astrocytoma (aHR 1.66 [95% CI 1.08–2.56] *P*-value = 0.020). Among cases of medulloblastoma, infants born small for gestational age showed a higher risk of death compared to those born appropriate size for age (aHR 3.12 [95% CI 1.56–6.24] *P*-value = 0.001). For ependymoma cases, children of mothers born in countries other than the U.S. or Mexico, had a higher risk of death (aHR 2.50 [95% CI 1.05–5.98] *P*-value = 0.039) (Table 5).

**Table 5:** Significant associations between maternal and perinatal factors and survival of pediatric astrocytoma, ependymoma, and medulloblastoma cases.

Maternal and perinatal characteristics	Astrocytoma						
	Cases	Unadjusted model			Adjusted model^a^		
		HR	95% CI	P-value^b^	HR	95% CI	P-value^b^
**Maternal race/ethnicity**							
Non-hispanic white	480 (49.5)	Reference			Reference		
Non-hispanic black	101 (10.4)	1.68	1.11–2.56	0.015	1.66	1.08–2.56	0.020
Hispanic	357 (36.8)	1.27	0.94–1.72	0.121	1.30	0.95–1.79	0.105
Other	32 (3.3)	1.89	0.98–3.64	0.056	1.98	0.99–3.95	0.054
Missing	0 (0.0)						
	**Ependymoma**						
**Maternal nativity**							
U.S. born	99 (63.1)	Reference			Reference		
Mexico	40 (25.5)	1.16	0.59–2.29	0.667	1.19	0.54–2.69	0.659
Other	17 (10.8)	2.11	0.96–4.66	0.065	2.50	1.05–5.98	0.039
Missing	1 (0.6)						
	**Medulloblastoma**						
**Size for gestational age**							
< 10th percentile	27 (12.4)	2.09	1.10–3.96	0.024	3.12	1.56–6.24	0.001
10th–90th percentile	164 (75.6)	Reference			Reference		
> 90th percentile	24 (11.1)	1.11	0.52–2.38	0.782	1.01	0.46–2.21	0.984
Missing	2 (0.9)						

^a^Adjusted for birth year, sex, maternal race/ethnicity, maternal education, tumor malignancy.

^b^Bonferroni corrected reference *P* values: 0.003 for an experiment-wide significance of 0.05.

**Figure 1:**
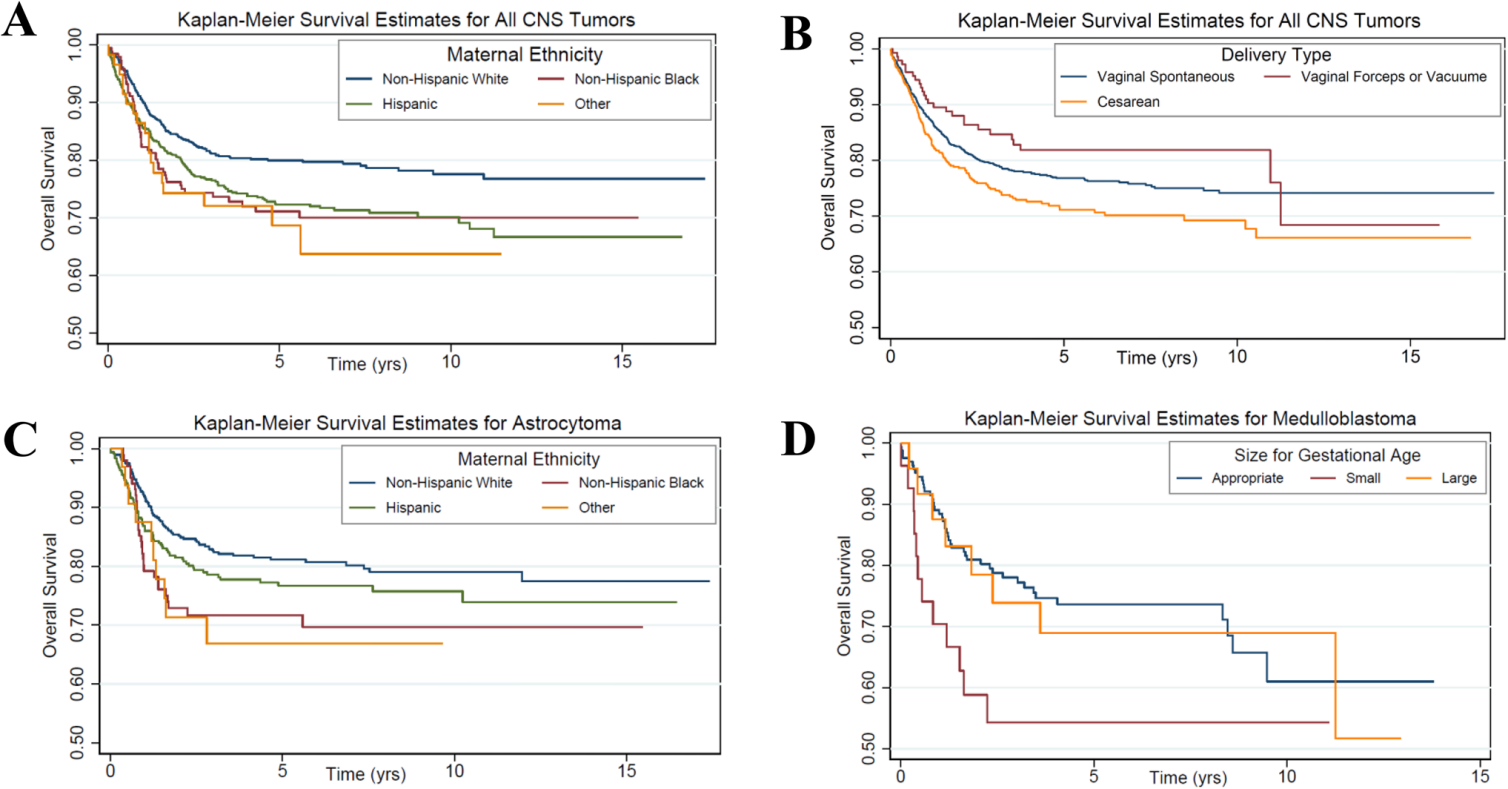
Kaplan–Meier estimates for overall survival in cases with pediatric central nervous system tumors according to maternal and perinatal factors. Overall survival based on Kaplan–Meier estimates for (**A**) all CNC tumor cases and maternal ethnicity (*P* = 0.002); (**B**) all CNC tumor cases and delivery type (*P* = 0.012); (**C**) astrocytoma cases and maternal ethnicity (*P* = 0.033); (**D**) medulloblastoma cases and size for gestational age (*P* = 0.069).

Figure 1 illustrates the overall survival based on Kaplan–Meier estimates. Among all pediatric CNS tumor cases, children of non-Hispanic white mothers had superior survival compared to children of mothers belong to any other ethnicity/race groups (*P*-value = 0.002). This was also true among astrocytoma cases, although the results were less pronounced (*P*-value = 0.033). Among all pediatric CNS tumor cases, infants born by instrument-assisted delivery experienced superior survival compared to those delivered vaginally or by cesarean (*P*-value = 0.012). However, among medulloblastoma cases, children born small for gestational age had worse survival compared to those born large or appropriate for gestational age, but the results were not statistically significant (*P*-value = 0.069). To assess for the potential effects of reverse causality, we performed a sensitivity analysis excluding children diagnosed with their brain tumor in the first year of life. The identified associations remained virtually unchanged, providing no evidence of reverse causality in these data.

Overall, we performed 15 testing procedures for each histological subtype. When the Bonferroni correction is applied, the reference *P*-value is 0.003 for an experiment-wide significance level of 0.05.

Corresponding non-significant findings are found in Supplementary Tables 1–8; where Supplementary Tables 1 and 5 summarizes the non-significant results for all CNS tumors, Supplementary Tables 2 and 6 summarizes the non-significant results for astrocytoma, Supplementary Tables 3 and 7 summarizes the non-significant results for ependymoma, and Supplementary Tables 4 and 8 summarizes the non-significant results for medulloblastoma.

## Discussion

In this large population-based study, we identified several maternal and perinatal factors associated with pediatric primary CNS tumor risk and survival. We observed racial/ethnic disparities in risk and survival of pediatric CNS tumors. Various delivery procedures were associated with both CNS tumor susceptibility and outcome; whereas, size for gestational age, gestational age, and infant sex were only associated with risk of CNS tumors in children. Additionally, some factors such as maternal education and maternal nativity were associated with risk and survival of specific histological subtypes of pediatric brain tumors. While some of these factors have been shown to be associated with pediatric CNS tumors in previous assessments, none are considered to be well-established factors for risk or survival of these malignancies^3–5^.

Although children of Hispanic and non-Hispanic black mothers had a decreased risk of developing CNS tumors, they exhibited a higher risk of death. Consistent with the findings of our study, the Central Brain Tumor Registry of the United States (CBTRUS), reported that the average annual age-adjusted incidence rate of pediatric CNS tumors is lower among Hispanic children compared to non-Hispanic children, as well as among black children compared to whites^1,2^. Additionally, survival differences by race for CNS tumors have been reported indicating poorer survival among non-Hispanic black and Hispanic patients^12–15^. The causes for these racial/ethnic differences in risk and survival of pediatric CNS tumors are not completely understood. However, differences could be due to genetic factors, differences in tumor biology, environmental and socioeconomic factors, and differences in access to health care, in general, and neuro-surgical and neuro-oncological services, specifically^16,17^.

In our study, infants delivered with vaginal forceps or vacuum showed a higher risk of developing CNS tumors, whereas those born by cesarean had a higher risk of death. An elevated risk of pediatric brain tumors associated with forceps or vacuum assisted vaginal delivery has been previously reported by two small studies^18,19^, but not in a third study^20^. There is evidence of associations between instrument-assisted delivery and increased risk of head injury^21,22^, which in turn is associated with an elevated risk of brain tumors in adults^23,24^. However, this topic merits further investigation for both adult and pediatric CNS tumors, and larger studies are warranted to replicate these findings. Further, a positive association between cesarean delivery and risk of pediatric malignancies, other than brain tumors, was previously reported^25–27^. The biological mechanisms underlying our findings of worse survival among children diagnosed with CNS tumors who were delivered by cesarean are unclear. The effects of cesarean delivery on risk and survival for pediatric CNS tumors could vary depending on whether it is performed before (elective cesarean delivery) or during labor (emergency cesarean delivery). However, the data available for the current study did not provide the information to distinguish these types of cesarean delivery.

There is evidence in the literature to suggest high birth weight and pre-term birth are associated with development of brain tumors in children^3,4^. In this study, the risk effect of high birth weight on pediatric CNS tumor development did not remain significant after controlling for potential confounders. However, being born large for gestational age and pre-term birth were robustly associated with an elevated risk of pediatric CNS tumors. It has been suggested that size for gestational age as an indicator for fatal growth is a better predictor for pediatric malignancies than birth weight independently. The mechanisms underlying these observed associations are unclear. However, epigenetic alterations in pre-term delivery, as well as higher levels of insulin-like growth factor-1, which is involved in brain ontogenesis and carcinogenesis and higher rate of cell divisions due to greater number of cells in children with high birth weight could explain these observations^28–30^.

Maternal education can be considered as a proxy for maternal socioeconomic status. There is evidence of a positive association between high parental socioeconomic status and risk of pediatric brain tumors in offspring^3,4,31^. Thus, our finding of increased risk of ependymoma associated with high maternal education is consistent with the results of previous assessments. Additionally, the finding of lower risk of developing pediatric CNS tumors among girls compared to boys is uniform to past reports^1,3,4^. The underlying mechanisms of these observations are largely unknown.

The primary strengths of the present study are that it is population-based and represents one of the largest studies, considering the rarity of the disease, to investigate the associations between maternal and perinatal characteristics and pediatric CNS tumors. Utilizing population-based registry data for both case and control identification, as well as for obtaining information about maternal, perinatal, and potential confounding factors, limits selection and recall bias. Additionally, this study assesses an ethnically and racially diverse population providing a unique ability to identify subgroups with higher susceptibility to or worse outcome from CNS tumors.

This study has some limitations. Although this is one of largest studies performed on pediatric CNS tumors, the sample size was still limited to investigate the associations within rare histological subtypes. Hence, replication studies are needed to confirm these findings and to detect risk and outcome predictors for rare histological subtypes of pediatric CNS tumors using larger sample sizes. Further, the cancer registry did not have information on co-existence of birth defects nor early-life exposure to ionizing radiation, two established risk factors for pediatric CNS tumors. Therefore, we were unable to assess these potential confounders in our analyses. There is also a small possibility that children classified as controls in this analysis have moved out of state and had cancer diagnoses that would not be registered in the TCR. However, due to rarity of childhood cancer, particularly CNS tumors, the effect of differential misclassification of exposure on our findings is likely to be low. As the study period included births through the year 2011 and follow-up was complete through December 2012, a proportion of the cohort, therefore, was not followed for 16 years to ascertain incident cases. However, in the analyses for etiological factors, we frequency-matched controls to cases on year of birth. Therefore, the potential effect of a shorter ascertainment period for cases diagnosed in more recent years would be minimized. Additionally, the average follow-up time for survival was 5.7 years, and the survival time for 75% of the deceased individuals was < 1.7 years, indicating only a small proportion of potentially undetected deceased cases. Additionally, we could not consider the potential differences in access to neuro-surgical and neuro-oncological services, which could influence survival among cases. Also, information on the molecular classification of the histological subtypes of brain tumors was not available in the cancer registry and therefore it is hard to interpret clinical associations of these findings based on the molecular subgroups of pediatric brain tumors.

In conclusion, in this large registry-based study, several maternal and perinatal factors were associated with risk and survival of podiatric CNS tumors, including maternal race/ethnicity, infant sex, gestational age, size for gestational age, and mode of delivery. Utilizing large available databases including high-quality registry data is beneficial to identifying risk and outcome predictors for such clinically significant malignancies, which has potential public health and clinical implications. Incorporating the findings of this study with knowledge on molecular characteristics of pediatric CNS tumor subtypes might lead to the development of novel comprehensive risk and outcome prediction models which have been lacking for the management of pediatric brain tumors.

## Supplementary Information

Supplementary Table 1.

Supplementary Table 2.

Supplementary Table 3.

Supplementary Table 4.

Supplementary Table 5.

Supplementary Table 6.

Supplementary Table 7.

Supplementary Table 8.

## Data Availability

The datasets used and/or analyzed during the current study are available on reasonable requests.
