# Supplementary material for: Maternal and perinatal factors are associated with risk of pediatric central nervous system tumors and poorer survival after diagnosis
Source: Sci Rep. 2021 May 17;11:10410. doi: 10.1038/s41598-021-88385-3 (PMC8129132; doi:10.1038/s41598-021-88385-3)
Supplement: Supplementary file 1 — Supplementary Table 1. [file 41598_2021_88385_MOESM1_ESM.docx]

Supplementary Table 1. Non-significant associations between maternal and perinatal factors and risk of pediatric central nervous system tumors

| **Maternal and perinatal characteristics** | **All Nervous system tumors** | | | | | | | |
| --- | --- | --- | --- | --- | --- | --- | --- | --- |
|  | **Cases** | **Controls** | **Unadjusted model** | | | **Adjusted model ^a^** | | |
|  |  |  | **OR** | **95%CI** | **p-value ^b^** | **OR** | **95%CI** | **p-value ^b^** |
| **Maternal age** |  |  |  |  |  |  |  |  |
| <25 | 788 (40.8) | 8,290 (42.9) | 0.91 | 0.81-1.03 | 0.126 | 0.92 | 0.81-1.04 | 0.169 |
| 25-29 | 530 (27.4) | 5,095 (26.4) | Reference | | | Reference | | |
| 30-34 | 403 (20.8) | 3,838 (19.8) | 1.01 | 0.88-1.16 | 0.893 | 1.00 | 0.87-1.15 | 0.992 |
| ≥35 | 213 (11.0) | 2,117 (10.9) | 0.97 | 0.82-1.14 | 0.696 | 0.95 | 0.80-1.13 | 0.568 |
| Continuous |  |  | 1.00 | 0.99-1.01 | 0.278 | 1.00 | 0.99-1.01 | 0.611 |
| Missing | 0 (0.0) | 0 (0.0) |  |  |  |  |  |  |
| **Maternal education** |  |  |  |  |  |  |  |  |
| < High school | 547 (28.3) | 5,884 (30.4) | 0.88 | 0.78-0.99 | 0.038 | 0.90 | 0.79-1.02 | 0.102 |
| High school | 603 (31.2) | 5,706 (29.5) | Reference | | | Reference | | |
| > High school | 761 (39.3) | 7,524 (38.9) | 0.96 | 0.86-1.07 | 0.444 | 0.93 | 0.83-1.05 | 0.234 |
| Missing | 23 (1.2) | 226 (1.2) |  |  |  |  |  |  |
| **Maternal nativity** |  |  |  |  |  |  |  |  |
| U.S. born | 1,444 (74.7) | 14,222 (73.5) | Reference | |  | Reference | | |
| Mexico | 343 (17.7) | 3,512 (18.2) | 0.96 | 0.85-1.09 | 0.537 | 1.07 | 0.92-1.25 | 0.366 |
| Other | 138 (7.1) | 1,559 (8.1) | 0.87 | 0.73-1.05 | 0.140 | 0.95 | 0.76-1.17 | 0.613 |
| Missing | 9 (0.5) | 47 (0.2) |  |  |  |  |  |  |
| **Residence on Mexican border** |  |  |  |  |  |  |  |  |
| No | 1,720 (88.9) | 17,120 (88.5) | Reference | | Reference | | | |
| Yes | 214 (11.1) | 2,220 (11.5) | 0.96 | 0.83-1.11 | 0.586 | 1.00 | 0.84-1.16 | 0.914 |
| Missing | 0 (0.0) | 0 (0.0) |  |  |  |  |  |  |
| **Maternal residency** |  |  |  |  |  |  |  |  |
| Urban | 1,636 (84.6) | 16,302 (84.3) | Reference | | | Reference | | |
| Rural | 85 (4.4) | 832 (4.3) | 1.02 | 0.81-1.28 | 0.879 | 1.00 | 0.79-1.26 | 0.981 |
| Missing | 213 (11.0) | 2,206 (11.4) |  |  |  |  |  |  |
| **Plurality** |  |  |  |  |  |  |  |  |
| Singleton | 1,866 (96.5) | 18,803 (97.2) | Reference | | | Reference | | |
| ≥2 | 68 (3.5) | 537 (2.8) | 1.28 | 0.99-1.65 | 0.063 | 1.26 | 0.97-1.63 | 0.084 |
| Missing | 0 (0.0) | 0 (0.0) |  |  |  |  |  |  |
| **Birth order** |  |  |  |  |  |  |  |  |
| 1st | 1,501 (77.6) | 14,943 (77.3) | Reference | | | Reference | | |
| 2nd | 283 (14.6) | 2,846 (14.7) | 0.99 | 0.87-1.13 | 0.882 | 0.98 | 0.86-1.13 | 0.821 |
| ≥3rd | 107 (5.6) | 1,151 (5.9) | 0.93 | 0.75-1.14 | 0.459 | 0.92 | 0.75-1.13 | 0.411 |
| Continuous |  |  | 0.97 | 0.90-1.04 | 0.355 | 0.96 | 0.89-1.03 | 0.285 |
| Missing | 43 (2.2) | 400 (2.1) |  |  |  |  |  |  |
| **Maternal BMI ^c^** |  |  |  |  |  |  |  |  |
| <18.5 | 17 (3.9) | 207 (4.7) | 0.79 | 0.47-1.32 | 0.371 | 0.82 | 0.49-1.36 | 0.437 |
| 18.5-24.9 | 221 (50.7) | 2,129 (48.8) | Reference | | | Reference | | |
| 25-29.9 | 101 (23.2) | 1,107 (25.4) | 0.88 | 0.69-1.12 | 0.305 | 0.89 | 0.69-1.14 | 0.365 |
| ≥30 | 93 (21.4) | 888 (20.4) | 1.01 | 0.78-1.30 | 0.946 | 0.99 | 0.77-1.29 | 0.960 |
| Continuous |  |  | 0.99 | 0.98-1.02 | 0.899 | 0.99 | 0.98-1.01 | 0.807 |
| Missing | 4 (0.9) | 29 (0.7) |  |  |  |  |  |  |
| **Maternal smoking** |  |  |  |  |  |  |  |  |
| No | 1,787 (92.4) | 17,912 (92.6) | Reference | | | Reference | | |
| Yes | 123 (6.4) | 1,188 (6.1) | 1.04 | 0.86-1.26 | 0.705 | 0.98 | 0.80-1.19 | 0.821 |
| Missing | 24 (1.2) | 240 (1.3) |  |  |  |  |  |  |

^a^ Adjusted for birth year, sex, maternal race/ethnicity, and maternal education

^b^ Bonferroni corrected reference *P values*: 0.003 for an experiment-wide significance of 0.05

^c^ Pre-pregnancy maternal body mass index (BMI) data collection began in 2005
