# Supplementary material for: Maternal and perinatal factors are associated with risk of pediatric central nervous system tumors and poorer survival after diagnosis
Source: Sci Rep. 2021 May 17;11:10410. doi: 10.1038/s41598-021-88385-3 (PMC8129132; doi:10.1038/s41598-021-88385-3)
Supplement: Supplementary file 2 — Supplementary Table 2. [file 41598_2021_88385_MOESM2_ESM.docx]

Supplementary Table 2. Non-significant associations between maternal and perinatal factors and risk of astrocytoma in children

| **Maternal and perinatal characteristics** | **Astrocytoma** | | | | | | | |
| --- | --- | --- | --- | --- | --- | --- | --- | --- |
|  | **Cases** | **Controls** | **Unadjusted model** | | | **Adjusted model ^a^** | | |
|  |  |  | **OR** | **95%CI** | **p-value ^b^** | **OR** | **95%CI** | **p-value ^b^** |
| **Maternal age** |  |  |  |  |  |  |  |  |
| <25 | 392 (40.4) | 4,126 (42.5) | 0.94 | 0.79-1.10 | 0.423 | 0.99 | 0.83-1.17 | 0.876 |
| 25-29 | 260 (26.8) | 2,559 (26.4) | Reference | | | Reference | | |
| 30-34 | 194 (20.0) | 1,975 (20.4) | 0.97 | 0.79-1.17 | 0.734 | 0.93 | 0.76-1.13 | 0.452 |
| ≥35 | 124 (12.8) | 1,040 (10.7) | 1.17 | 0.94-1.47 | 0.165 | 1.14 | 0.90-1.43 | 0.274 |
| Continuous |  |  | 1.01 | 0.99-1.02 | 0.067 | 1.00 | 0.99-1.02 | 0.532 |
| Missing | 0 (0.0) | 0 (0.0) |  |  |  |  |  |  |
| **Maternal education** |  |  |  |  |  |  |  |  |
| < High school | 248 (25.6) | 2,929 (30.2) | 0.83 | 0.69-0.99 | 0.035 | 0.89 | 0.74-1.06 | 0.198 |
| High school | 296 (30.5) | 2,892 (29.8) | Reference | | | Reference | | |
| > High school | 413 (42.6) | 3,756 (38.7) | 1.07 | 0.92-1.26 | 0.371 | 1.03 | 0.88-1.21 | 0.737 |
| Missing | 13 (1.3) | 123 (1.3) |  |  |  |  |  |  |
| **Maternal nativity** |  |  |  |  |  |  |  |  |
| U.S. born | 750 (77.3) | 7,119 (73.4) | Reference | | | Reference | | |
| Mexico | 143 (14.8) | 1,756 (18.1) | 0.77 | 0.64-0.93 | 0.007 | 0.98 | 0.78-1.23 | 0.854 |
| Other | 73 (7.5) | 795 (8.2) | 0.87 | 0.68-1.12 | 0.284 | 0.96 | 0.71-1.29 | 0.778 |
| Missing | 4 (0.4) | 30 (0.3) |  |  |  |  |  |  |
| **Residence on Mexican border** |  |  |  |  |  |  |  |  |
| No | 883 (91.0) | 8,605 (88.7) | Reference | | | Reference | | |
| Yes | 87 (9.0) | 1,095 (11.3) | 0.77 | 0.62-0.97 | 0.029 | 0.88 | 0.69-1.13 | 0.329 |
| Missing | 0 (0.0) | 0 (0.0) |  |  |  |  |  |  |
| **Maternal residency** |  |  |  |  |  |  |  |  |
| Urban | 831 (85.7) | 8,199 (84.5) | Reference | | | Reference | | |
| Rural | 45 (4.6) | 403 (4.2) | 1.10 | 0.80-1.51 | 0.548 | 1.06 | 0.77-1.46 | 0.709 |
| Missing | 94 (9.7) | 1,098 (11.3) |  |  |  |  |  |  |
| **Infant sex** |  |  |  |  |  |  |  |  |
| Male | 496 (51.1) | 4,905 (50.6) | Reference | | | Reference | | |
| Female | 474 (48.9) | 4,795 (49.4) | 0.98 | 0.86-1.12 | 0.736 | 0.97 | 0.85-1.11 | 0.687 |
| Missing | 0 (0.0) | 0 (0.0) |  |  |  |  |  |  |
| **Plurality** |  |  |  |  |  |  |  |  |
| Singleton | 934 (96.3) | 9,432 (97.2) | Reference | | | Reference | | |
| ≥2 | 36 (3.7) | 268 (2.8) | 1.36 | 0.95-1.93 | 0.092 | 1.32 | 0.92-1.88 | 0.129 |
| Missing | 0 (0.0) | 0 (0.0) |  |  |  |  |  |  |
| **Birth order** |  |  |  |  |  |  |  |  |
| 1st | 743 (76.6) | 7,466 (77.0) | Reference | | | Reference | | |
| 2nd | 143 (14.7) | 1,452 (15.0) | 0.99 | 0.82-1.19 | 0.913 | 0.98 | 0.81-1.18 | 0.812 |
| ≥3rd | 61 (6.3) | 584 (6.0) | 1.05 | 0.79-1.38 | 0.730 | 1.01 | 0.77-1.34 | 0.923 |
| Continuous |  |  | 0.99 | 0.91-1.09 | 0.864 | 0.98 | 0.89-1.07 | 0.630 |
| Missing | 23 (2.4) | 198 (2.0) |  |  |  |  |  |  |
| **Size for gestational age** |  |  |  |  |  |  |  |  |
| <10^th^ percentile | 115 (11.9) | 1,281 (13.2) | 0.91 | 0.74-1.11 | 0.353 | 0.89 | 0.73-1.10 | 0.301 |
| 10^th^_-_90^th^ percentile | 741 (76.4) | 7,490 (77.2) | Reference | | | Reference | | |
| >90^th^ percentile | 98 (10.1) | 825 (8.5) | 1.20 | 0.96-1.50 | 0.107 | 1.19 | 0.96-1.49 | 0.112 |
| Missing | 16 (1.6) | 104 (1.1) |  |  |  |  |  |  |
| **Birth weight (g)** |  |  |  |  |  |  |  |  |
| <2500 | 70 (7.2) | 723 (7.4) | 0.98 | 0.76-1.26 | 0.873 | 0.99 | 0.76-1.28 | 0.944 |
| 2500-3999 | 815 (84.0) | 8,244 (85.0) | Reference | | | Reference | | |
| ≥4000 | 85 (8.8) | 724 (7.5) | 1.19 | 0.94-1.50 | 0.153 | 1.17 | 0.92-1.48 | 0.203 |
| Continuous |  |  | 1.00 | 0.99-1.00 | 0.087 | 1.00 | 0.99-1.00 | 0.178 |
| Missing | 0 (0.0) | 9 (0.1) |  |  |  |  |  |  |
| **Maternal BMI ^c^** |  |  |  |  |  |  |  |  |
| <18.5 | 8 (3.8) | 86 (4.2) | 0.95 | 0.45-2.01 | 0.884 | 0.98 | 0.46-2.09 | 0.962 |
| 18.5-24.9 | 98 (47.6) | 996 (48.3) | Reference | | | Reference | | |
| 25-29.9 | 50 (24.3) | 526 (25.5) | 0.97 | 0.68-1.38 | 0.850 | 0.98 | 0.69-1.41 | 0.921 |
| ≥30 | 49 (23.8) | 442 (21.5) | 1.13 | 0.79-1.62 | 0.517 | 1.12 | 0.78-1.62 | 0.537 |
| Continuous |  |  | 1.01 | 0.99-1.03 | 0.346 | 1.01 | 0.99-1.04 | 0.353 |
| Missing | 1 (0.5) | 10 (0.5) |  |  |  |  |  |  |
| **Maternal smoking** |  |  |  |  |  |  |  |  |
| No | 882 (91.0) | 8,961 (92.4) | Reference | | | Reference | | |
| Yes | 73 (7.5) | 613 (6.3) | 1.21 | 0.94-1.56 | 0.139 | 1.11 | 0.85-1.45 | 0.432 |
| Missing | 15 (1.5) | 126 (1.3) |  |  |  |  |  |  |

^a^ Adjusted for birth year, sex, maternal race/ethnicity, and maternal education

^b^ Bonferroni corrected reference *P values*: 0.003 for an experiment-wide significance of 0.05

^c^ Pre-pregnancy maternal body mass index (BMI) data collection began in 2005
