# Supplementary material for: Maternal and perinatal factors are associated with risk of pediatric central nervous system tumors and poorer survival after diagnosis
Source: Sci Rep. 2021 May 17;11:10410. doi: 10.1038/s41598-021-88385-3 (PMC8129132; doi:10.1038/s41598-021-88385-3)
Supplement: Supplementary file 3 — Supplementary Table 3. [file 41598_2021_88385_MOESM3_ESM.docx]

Supplementary Table 3. Non-significant associations between maternal and perinatal factors and risk of ependymoma in children

| **Maternal and perinatal characteristics** | **Ependymoma** | | | | | | | |
| --- | --- | --- | --- | --- | --- | --- | --- | --- |
|  | **Cases** | **Controls** | **Unadjusted model** | | | **Adjusted model ^a^** | | |
|  |  |  | **OR** | **95%CI** | **p-value ^b^** | **OR** | **95%CI** | **p-value ^b^** |
| **Maternal race/ethnicity** |  |  |  |  |  |  |  |  |
| Non-Hispanic White | 61 (38.8) | 623 (39.7) | Reference | | | Reference | | |
| Non-Hispanic Black | 10 (6.4) | 177 (11.3) | 0.58 | 0.29-1.15 | 0.118 | 0.53 | 0.27-1.07 | 0.076 |
| Hispanic | 82 (52.2) | 703 (44.8) | 1.19 | 0.84-1.69 | 0.325 | 1.06 | 0.72-1.57 | 0.763 |
| Other | 4 (2.6) | 67 (4.2) | 0.61 | 0.21-1.73 | 0.352 | 0.71 | 0.25-2.02 | 0.517 |
| Missing | 0 (0.0) | 0 (0.0) |  |  |  |  |  |  |
| **Maternal age** |  |  |  |  |  |  |  |  |
| <25 | 69 (43.9) | 682 (43.4) | 1.07 | 0.71-1.62 | 0.757 | 0.96 | 0.62-1.48 | 0.841 |
| 25-29 | 38 (24.2) | 401 (25.5) | Reference | | | Reference | | |
| 30-34 | 35 (22.3) | 326 (20.8) | 1.13 | 0.69-83 | 0.612 | 1.27 | 0.78-2.07 | 0.341 |
| ≥35 | 15 (9.6) | 161 (10.3) | 0.98 | 0.53-84 | 0.958 | 1.02 | 0.53-1.95 | 0.954 |
| Continuous |  |  | 0.99 | 0.96-1.02 | 0.515 | 1.00 | 0.97-1.03 | 0.875 |
| Missing | 0 (0.0) | 0 (0.0) |  |  |  |  |  |  |
| **Residence on Mexican border** |  |  |  |  |  |  |  |  |
| No | 128 (81.5) | 1,386 (88.3) | Reference | | | Reference | | |
| Yes | 29 (18.5) | 184 (11.7) | 1.71 | 1.11-2.63 | 0.015 | 1.52 | 0.94-2.45 | 0.085 |
| Missing | 0 (0.0) | 0 (0.0) |  |  |  |  |  |  |
| **Maternal residency** |  |  |  |  |  |  |  |  |
| Urban | 132 (84.1) | 1,334 (85.0) | Reference | | | Reference | | |
| Rural | 6 (3.8) | 60 (3.8) | 1.01 | 0.43-2.38 | 0.981 | 0.95 | 0.40-2.27 | 0.915 |
| Missing | 19 (12.1) | 176 (11.2) |  |  |  |  |  |  |
| **Infant sex** |  |  |  |  |  |  |  |  |
| Male | 90 (57.3) | 781 (49.8) | Reference | | | Reference | | |
| Female | 67 (42.7) | 789 (50.2) | 0.74 | 0.53-1.03 | 0.071 | 0.72 | 0.51-1.01 | 0.055 |
| Missing | 0 (0.0) | 0 (0.0) |  |  |  |  |  |  |
| **Plurality** |  |  |  |  |  |  |  |  |
| Singleton | 152 (96.8) | 1,529 (97.4) | Reference | | | Reference | | |
| ≥2 | 5 (3.2) | 41 (2.6) | 1.23 | 0.48-3.15 | 0.671 | 1.23 | 0.47-3.18 | 0.675 |
| Missing | 0 (0.0) | 0 (0.0) |  |  |  |  |  |  |
| **Birth order** |  |  |  |  |  |  |  |  |
| 1st | 128 (81.5) | 1,191 (75.9) | Reference | | | Reference | | |
| 2nd | 19 (12.1) | 257 (16.3) | 0.69 | 0.42-1.13 | 0.143 | 0.73 | 0.44-1.21 | 0.228 |
| ≥3rd | 7 (4.5) | 97 (6.2) | 0.67 | 0.31-1.48 | 0.322 | 0.76 | 0.34-1.68 | 0.500 |
| Continuous |  |  | 0.82 | 0.62-1.08 | 0.164 | 0.86 | 0.66-1.13 | 0.291 |
| Missing | 3 (1.9) | 25 (1.6) |  |  |  |  |  |  |
| **Size for gestational age** |  |  |  |  |  |  |  |  |
| <10^th^ percentile | 25 (15.9) | 228 (14.5) | 1.10 | 0.69-1.74 | 0.675 | 1.15 | 0.72-1.82 | 0.557 |
| 10^th^_-_90^th^ percentile | 118 (75.2) | 1,186 (75.6) | Reference | | | Reference | | |
| >90^th^ percentile | 13 (8.3) | 134 (8.5) | 0.98 | 0.54-1.78 | 0.934 | 1.00 | 0.55-1.84 | 0.989 |
| Missing | 1 (0.6) | 22 (1.4) |  |  |  |  |  |  |
| **Delivery type** |  |  |  |  |  |  |  |  |
| Vaginal spontaneous | 101 (64.3) | 1,047 (66.8) | Reference | | | Reference | | |
| Vaginal forceps or vacuum | 11 (7.0) | 91 (5.8) | 1.25 | 0.65-2.42 | 0.502 | 1.25 | 0.64-2.45 | 0.507 |
| Cesarean | 45 (28.7) | 430 (27.4) | 1.08 | 0.75-1.57 | 0.665 | 1.05 | 0.72-1.54 | 0.795 |
| Missing | 0 (0.0) | 2 (0.1) |  |  |  |  |  |  |
| **Birth weight (g)** |  |  |  |  |  |  |  |  |
| <2500 | 16 (10.2) | 113 (7.2) | 1.44 | 0.83-2.51 | 0.196 | 1.44 | 0.81-2.57 | 0.211 |
| 2500-3999 | 130 (82.8) | 1,323 (84.3) | Reference | | | Reference | | |
| ≥4000 | 11 (7.0) | 134 (8.5) | 0.84 | 0.44-1.59 | 0.582 | 0.66 | 0.33-1.34 | 0.247 |
| Continuous |  |  | 0.99 | 0.99-1.00 | 0.951 | 0.99 | 0.99-1.00 | 0.672 |
| Missing | 0 (0.0) | 0 (0.0) |  |  |  |  |  |  |
| **Maternal smoking** |  |  |  |  |  |  |  |  |
| No | 147 (93.6) | 1,467 (93.4) | Reference | | | Reference | | |
| Yes | 10 (6.4) | 84 (5.4) | 1.19 | 0.60-2.34 | 0.618 | 1.18 | 0.58-2.39 | 0.647 |
| Missing | 0 (0.0) | 19 (1.2) |  |  |  |  |  |  |

^a^ Adjusted for birth year, sex, maternal race/ethnicity, and maternal education

^b^ Bonferroni corrected reference *P values*: 0.003 for an experiment-wide significance of 0.05
