# Supplementary material for: Maternal and perinatal factors are associated with risk of pediatric central nervous system tumors and poorer survival after diagnosis
Source: Sci Rep. 2021 May 17;11:10410. doi: 10.1038/s41598-021-88385-3 (PMC8129132; doi:10.1038/s41598-021-88385-3)
Supplement: Supplementary file 4 — Supplementary Table 4. [file 41598_2021_88385_MOESM4_ESM.docx]

Supplementary Table 4. Non-significant associations between maternal and perinatal factors and risk of medulloblastoma in children

| **Maternal and perinatal characteristics** | **Medulloblastoma** | | | | | | | |
| --- | --- | --- | --- | --- | --- | --- | --- | --- |
|  | **Cases** | **Controls** | **Unadjusted model** | | | **Adjusted model ^a^** | | |
|  |  |  | **OR** | **95%CI** | **p-value ^b^** | **OR** | **95%CI** | **p-value ^b^** |
| **Maternal race/ethnicity** |  |  |  |  |  |  |  |  |
| Non-Hispanic White | 98 (45.2) | 935 (43.1) | Reference | | | Reference | | |
| Non-Hispanic Black | 17 (7.8) | 257 (11.8) | 0.63 | 0.37-1.08 | 0.091 | 0.65 | 0.38-1.12 | 0.123 |
| Hispanic | 96 (44.2) | 904 (41.7) | 1.01 | 0.75-1.36 | 0.931 | 0.99 | 0.72-1.39 | 0.998 |
| Other | 6 (2.8) | 74 (3.4) | 0.77 | 0.33-1.82 | 0.557 | 0.84 | 0.36-1.99 | 0.696 |
| Missing | 0 (0.0) | 0 (0.0) |  |  |  |  |  |  |
| **Maternal age** |  |  |  |  |  |  |  |  |
| <25 | 79 (36.4) | 897 (41.4) | 0.84 | 0.59-1.19 | 0.339 | 0.86 | 0.59-1.23 | 0.403 |
| 25-29 | 63 (29.0) | 604 (27.8) | Reference | | | Reference | | |
| 30-34 | 52 (24.0) | 417 (19.2) | 1.19 | 0.81-1.76 | 0.367 | 1.21 | 0.81-1.81 | 0.340 |
| ≥35 | 23 (10.6) | 252 (11.6) | 0.88 | 0.53-1.44 | 0.600 | 0.91 | 0.55-1.52 | 0.726 |
| Continuous |  |  | 1.01 | 0.99-1.03 | 0.552 | 1.01 | 0.98-1.03 | 0.633 |
| Missing | 0 (0.0) | 0 (0.0) |  |  |  |  |  |  |
| **Maternal education** |  |  |  |  |  |  |  |  |
| < High school | 63 (29.0) | 642 (29.6) | 0.96 | 0.67-1.39 | 0.839 | 0.96 | 0.66-1.41 | 0.834 |
| High school | 64 (29.5) | 628 (28.9) | Reference | | | Reference | | |
| > High school | 87 (40.1) | 871 (40.1) | 0.98 | 0.69-1.38 | 0.908 | 0.99 | 0.69-1.39 | 0.939 |
| Missing | 3 (1.4) | 29 (1.3) |  |  |  |  |  |  |
| **Residence on Mexican border** |  |  |  |  |  |  |  |  |
| No | 188 (86.6) | 1,926 (88.8) | Reference | | | Reference | | |
| Yes | 29 (13.4) | 244 (11.2) | 1.22 | 0.81-1.84 | 0.350 | 1.16 | 0.74-1.82 | 0.515 |
| Missing | 0 (0.0) | 0 (0.0) |  |  |  |  |  |  |
| **Maternal residency** |  |  |  |  |  |  |  |  |
| Urban | 179 (82.5) | 1,808 (83.3) | Reference | | | Reference | | |
| Rural | 10 (4.6) | 98 (4.5) | 1.03 | 0.53-2.01 | 0.929 | 1.04 | 0.53-2.05 | 0.903 |
| Missing | 28 (12.9) | 264 (12.2) |  |  |  |  |  |  |
| **Plurality** |  |  |  |  |  |  |  |  |
| Singleton | 212 (97.7) | 2,115 (97.5) | Reference | | | Reference | | |
| ≥2 | 5 (2.3) | 55 (2.5) | 0.91 | 0.36-2.29 | 0.836 | 0.98 | 0.39-2.49 | 0.969 |
| Missing | 0 (0.0) | 0 (0.0) |  |  |  |  |  |  |
| **Size for gestational age** |  |  |  |  |  |  |  |  |
| <10^th^ percentile | 27 (12.4) | 284 (13.1) | 0.96 | 0.63-1.47 | 0.847 | 0.92 | 0.59-1.42 | 0.699 |
| 10^th^_-_90^th^ percentile | 164 (75.6) | 1,654 (76.2) | Reference | | | Reference | | |
| >90^th^ percentile | 24 (11.1) | 208 (9.6) | 1.16 | 0.74-1.83 | 0.511 | 1.17 | 0.74-1.84 | 0.509 |
| Continuous |  |  |  |  |  |  |  |  |
| Missing | 2 (0.9) | 24 (1.1) |  |  |  |  |  |  |
| **Gestational age** |  |  |  |  |  |  |  |  |
| <37 weeks | 30 (13.8) | 249 (11.4) | 1.23 | 0.82-1.86 | 0.314 | 1.23 | 0.81-1.86 | 0.343 |
| 37-41 weeks | 179 (82.5) | 1,833 (84.5) | Reference | | | Reference | | |
| ≥42 | 6 (2.8) | 64 (3.0) | 0.96 | 0.41-2.25 | 0.925 | 0.99 | 0.42-2.35 | 0.991 |
| Continuous |  |  | 0.98 | 0.92-1.03 | 0.430 | 0.98 | 0.93-1.04 | 0.511 |
| Missing | 2 (0.9) | 24 (1.1) |  |  |  |  |  |  |
| **Delivery type** |  |  |  |  |  |  |  |  |
| Vaginal spontaneous | 140 (64.5) | 1,418 (65.4) | Reference | | | Reference | | |
| Vaginal forceps or vacuum | 11 (5.1) | 128 (5.9) | 0.87 | 0.46-1.65 | 0.671 | 0.86 | 0.45-1.63 | 0.636 |
| Cesarean | 66 (30.4) | 622 (28.6) | 1.07 | 0.79-1.46 | 0.646 | 1.04 | 0.76-1.42 | 0.800 |
| Missing | 0 (0.0) | 2 (0.1) |  |  |  |  |  |  |
| **Birth weight (g)** |  |  |  |  |  |  |  |  |
| <2500 | 18 (8.3) | 175 (8.1) | 1.06 | 0.64-1.77 | 0.817 | 1.04 | 0.61-1.75 | 0.894 |
| 2500-3999 | 176 (81.1) | 1,817 (83.7) | Reference | | | Reference | | |
| ≥4000 | 23 (10.6) | 176 (8.1) | 1.35 | 0.85-2.14 | 0.203 | 1.23 | 0.77-1.97 | 0.391 |
| Continuous |  |  | 1.00 | 0.99-1.00 | 0.359 | 1.00 | 0.99-1.00 | 0.567 |
| Missing | 0 (0.0) | 2 (0.1) |  |  |  |  |  |  |
| **Maternal BMI ^c^** |  |  |  |  |  |  |  |  |
| <18.5 | 2 (4.9) | 24 (5.9) | 0.63 | 0.14-2.83 | 0.548 | 0.73 | 0.16-3.37 | 0.688 |
| 18.5-24.9 | 26 (63.4) | 197 (48.1) | Reference | | | Reference | | |
| 25-29.9 | 8 (19.5) | 108 (26.3) | 0.56 | 0.25-1.28 | 0.171 | 0.59 | 0.26-1.36 | 0.217 |
| ≥30 | 5 (12.2) | 80 (19.5) | 0.47 | 0.18-1.28 | 0.140 | 0.49 | 0.18-1.37 | 0.175 |
| Continuous |  |  | 0.97 | 0.91-1.03 | 0.258 | 0.97 | 0.91-1.03 | 0.287 |
| Missing | 0 (0.0) | 1 (0.2) |  |  |  |  |  |  |
| **Maternal smoking** |  |  |  |  |  |  |  |  |
| No | 205 (94.5) | 1,976 (91.0) | Reference | | | Reference | | |
| Yes | 9 (4.1) | 160 (7.4) | 0.54 | 0.27-1.08 | 0.081 | 0.52 | 0.25-1.05 | 0.069 |
| Missing | 3 (1.4) | 34 (1.6) |  |  |  |  |  |  |

^a^ Adjusted for birth year, sex, maternal race/ethnicity, and maternal education

^b^ Bonferroni corrected reference *P values*: 0.003 for an experiment-wide significance of 0.05

^c^ Pre-pregnancy maternal body mass index (BMI) data collection began in 2005
