# Supplementary material for: Maternal and perinatal factors are associated with risk of pediatric central nervous system tumors and poorer survival after diagnosis
Source: Sci Rep. 2021 May 17;11:10410. doi: 10.1038/s41598-021-88385-3 (PMC8129132; doi:10.1038/s41598-021-88385-3)
Supplement: Supplementary file 5 — Supplementary Table 5. [file 41598_2021_88385_MOESM5_ESM.docx]

Supplementary Table 5. Non-significant associations between maternal and perinatal factors and survival of pediatric central nervous system tumor cases

| **Maternal and perinatal characteristics** | **All Nervous system tumors** | | | | | | |
| --- | --- | --- | --- | --- | --- | --- | --- |
|  | **Cases** | **Unadjusted model** | | | **Adjusted model ^a^** | | |
|  |  | **HR** | **95%CI** | **p-value ^b^** | **HR** | **95%CI** | **p-value ^b^** |
| **Maternal age** |  |  |  |  |  |  |  |
| <25 | 788 (40.8) | 1.18 | 0.94-1.48 | 0.153 | 1.19 | 0.94-1.52 | 0.149 |
| 25-29 | 530 (27.4) | Reference | | | Reference | | |
| 30-34 | 403 (20.8) | 0.82 | 0.61-1.09 | 0.176 | 0.86 | 0.64-1.15 | 0.317 |
| ≥35 | 213 (11.0) | 1.15 | 0.83-1.58 | 0.402 | 1.09 | 0.79-1.52 | 0.587 |
| Continuous |  | 0.99 | 0.98-1.01 | 0.268 | 0.99 | 0.98-1.01 | 0.426 |
| Missing | 0 (0.0) |  |  |  |  |  |  |
| **Maternal education** |  |  |  |  |  |  |  |
| < High school | 547 (28.3) | 1.03 | 0.81-1.31 | 0.784 | 0.96 | 0.75-1.23 | 0.753 |
| High school | 603 (31.2) | Reference | | | Reference | | |
| > High school | 761 (39.3) | 0.98 | 0.78-1.22 | 0.831 | 0.99 | 0.79-1.25 | 0.984 |
| Missing | 23 (1.2) |  |  |  |  |  |  |
| **Maternal nativity** |  |  |  |  |  |  |  |
| U.S. born | 1,444 (74.7) | Reference | | | Reference | | |
| Mexico | 343 (17.7) | 1.18 | 0.93-1.49 | 0.165 | 1.02 | 0.76-1.36 | 0.896 |
| Other | 138 (7.1) | 0.95 | 0.65-1.37 | 0.770 | 0.85 | 0.55-1.33 | 0.481 |
| Missing | 9 (0.5) |  |  |  |  |  |  |
| **Residence on Mexican border** |  |  |  |  |  |  |  |
| No | 1,720 (88.9) | Reference | | | Reference | | |
| Yes | 214 (11.1) | 1.10 | 0.83-1.47 | 0.500 | 0.91 | 0.66-1.24 | 0.544 |
| Missing | 0 (0.0) |  |  |  |  |  |  |
| **Maternal residency** |  |  |  |  |  |  |  |
| Urban | 1,636 (84.6) | Reference | | | Reference | | |
| Rural | 85 (4.4) | 1.11 | 0.71-1.72 | 0.653 | 1.12 | 0.72-1.73 | 0.625 |
| Missing | 213 (11.0) |  |  |  |  |  |  |
| **Infant sex** |  |  |  |  |  |  |  |
| Male | 1,032 (53.4) | Reference | | | Reference | | |
| Female | 902 (46.6) | 0.96 | 0.80-1.16 | 0.702 | 0.98 | 0.82-1.19 | 0.864 |
| Missing | 0 (0.0) |  |  |  |  |  |  |
| **Plurality** |  |  |  |  |  |  |  |
| Singleton | 1,866 (96.5) | Reference | | | Reference | | |
| ≥2 | 68 (3.5) | 1.27 | 0.80-2.01 | 0.306 | 1.22 | 0.76-1.96 | 0.409 |
| Missing | 0 (0.0) |  |  |  |  |  |  |
| **Birth order** |  |  |  |  |  |  |  |
| 1st | 1,501 (77.6) | Reference | | | Reference | | |
| 2nd | 283 (14.6) | 1.23 | 0.96-1.56 | 0.103 | 1.19 | 0.93-1.53 | 0.154 |
| ≥3rd | 107 (5.6) | 0.63 | 0.38-1.04 | 0.070 | 0.61 | 0.36-1.02 | 0.061 |
| Continuous |  | 0.95 | 0.82-1.09 | 0.457 | 0.95 | 0.81-1.1 | 0.472 |
| Missing | 43 (2.2) |  |  |  |  |  |  |
| **Size for gestational age** |  |  |  |  |  |  |  |
| <10^th^ percentile | 253 (13.1) | 1.18 | 0.90-1.53 | 0.234 | 1.13 | 0.86-1.48 | 0.383 |
| 10^th^_-_90^th^ percentile | 1,453 (75.1) | Reference | | | Reference | | |
| >90^th^ percentile | 200 ( 10.3) | 0.92 | 0.67-1.26 | 0.609 | 0.94 | 0.68-1.29 | 0.721 |
| Missing | 28 (1.5) |  |  |  |  |  |  |
| **Gestational age** |  |  |  |  |  |  |  |
| <37 weeks | 252 (13.0) | 0.94 | 0.71-1.25 | 0.676 | 0.87 | 0.65-1.16 | 0.339 |
| 37-41 weeks | 1,599 (82.7) | Reference | | | Reference | | |
| ≥42 | 55 (2.8) | 1.27 | 0.76-2.13 | 0.362 | 1.24 | 0.74-2.08 | 0.424 |
| Continuous |  | 0.99 | 0.95-1.04 | 0.804 | 1.00 | 0.96-1.05 | 0.918 |
| Missing | 28 (1.5) |  |  |  |  |  |  |
| **Birth weight (g)** |  |  |  |  |  |  |  |
| <2500 | 166 (8.6) | 1.11 | 0.80-1.54 | 0.534 | 1.04 | 0.74-1.45 | 0.840 |
| 2500-3999 | 1,596 (82.5) | Reference | | | Reference | | |
| ≥4000 | 172 (8.9) | 0.90 | 0.65-1.26 | 0.551 | 0.92 | 0.65-1.30 | 0.640 |
| Continuous |  | 0.99 | 0.99-1.00 | 0.183 | 0.99 | 0.99-1.00 | 0.445 |
| Missing | 0 (0.0) |  |  |  |  |  |  |
| **Maternal BMI ^c^** |  |  |  |  |  |  |  |
| <18.5 | 17 (3.9) | 0.45 | 0.11-1.86 | 0.270 | 0.51 | 0.12-2.13 | 0.358 |
| 18.5-24.9 | 221 (50.7) | Reference | | | Reference | | |
| 25-29.9 | 101 (23.2) | 0.72 | 0.42-1.25 | 0.245 | 0.73 | 0.42-1.26 | 0.259 |
| ≥30 | 93 (21.4) | 0.76 | 0.43-1.34 | 0.343 | 0.73 | 0.41-1.29 | 0.279 |
| Continuous |  | 0.98 | 0.95-1.02 | 0.418 | 0.98 | 0.95-1.02 | 0.358 |
| Missing | 4 (0.9) |  |  |  |  |  |  |
| **Maternal smoking** |  |  |  |  |  |  |  |
| No | 1,787 (92.4) | Reference | | | Reference | | |
| Yes | 123 (6.4) | 1.22 | 0.86-1.73 | 0.264 | 1.29 | 0.89-1.84 | 0.169 |
| Missing | 24 (1.2) |  |  |  |  |  |  |

^a^ Adjusted for birth year, sex, maternal race/ethnicity, maternal education, and tumor malignancy

^b^ Bonferroni corrected reference *P values*: 0.003 for an experiment-wide significance of 0.05

^c^ Pre-pregnancy maternal body mass index (BMI) data collection began in 2005
