# Supplementary material for: Maternal and perinatal factors are associated with risk of pediatric central nervous system tumors and poorer survival after diagnosis
Source: Sci Rep. 2021 May 17;11:10410. doi: 10.1038/s41598-021-88385-3 (PMC8129132; doi:10.1038/s41598-021-88385-3)
Supplement: Supplementary file 6 — Supplementary Table 6. [file 41598_2021_88385_MOESM6_ESM.docx]

Supplementary Table 6. Non-significant associations between maternal and perinatal factors and survival of pediatric astrocytoma cases

| **Maternal and perinatal characteristics** | **Astrocytoma** | | | | | | |
| --- | --- | --- | --- | --- | --- | --- | --- |
|  | **Cases** | **Unadjusted model** | | | **Adjusted model ^a^** | | |
|  |  | **HR** | **95%CI** | **p-value ^b^** | **HR** | **95%CI** | **p-value ^b^** |
| **Maternal age** |  |  |  |  |  |  |  |
| <25 | 392 (40.4) | 1.08 | 0.78-1.50 | 0.654 | 1.05 | 0.74-1.49 | 0.803 |
| 25-29 | 260 (26.8) | Reference | | | Reference | | |
| 30-34 | 194 (20.0) | 0.69 | 0.44-1.06 | 0.092 | 0.72 | 0.46-1.12 | 0.142 |
| ≥35 | 124 (12.8) | 1.02 | 0.65-1.59 | 0.944 | 1.07 | 0.68-1.69 | 0.773 |
| Continuous |  | 0.99 | 0.97-1.01 | 0.361 | 0.99 | 0.97-1.02 | 0.757 |
| Missing | 0 (0.0) |  |  |  |  |  |  |
| **Maternal education** |  |  |  |  |  |  |  |
| < High school | 248 (25.6) | 1.11 | 0.78-1.59 | 0.560 | 1.06 | 0.74-1.52 | 0.755 |
| High school | 296 (30.5) | Reference | | | Reference | | |
| > High school | 413 (42.6) | 0.95 | 0.69-1.32 | 0.758 | 0.99 | 0.71-1.39 | 0.981 |
| Missing | 13 (1.3) |  |  |  |  |  |  |
| **Maternal nativity** |  |  |  |  |  |  |  |
| U.S. born | 750 (77.3) | Reference | | | Reference | | |
| Mexico | 143 (14.8) | 1.01 | 0.69-1.49 | 0.953 | 0.92 | 0.58-1.46 | 0.733 |
| Other | 73 (7.5) | 0.81 | 0.46-1.43 | 0.474 | 0.61 | 0.31-1.21 | 0.160 |
| Missing | 4 (0.4) |  |  |  |  |  |  |
| **Residence on Mexican border** |  |  |  |  |  |  |  |
| No | 883 (91.0) | Reference | | | Reference | | |
| Yes | 87 (9.0) | 0.72 | 0.42-1.24 | 0.238 | 0.60 | 0.34-1.08 | 0.087 |
| Missing | 0 (0.0) |  |  |  |  |  |  |
| **Maternal residency** |  |  |  |  |  |  |  |
| Urban | 831 (85.7) | Reference | | | Reference | | |
| Rural | 45 (4.6) | 0.91 | 0.46-1.77 | 0.776 | 0.97 | 0.49-1.89 | 0.921 |
| Missing | 94 (9.7) |  |  |  |  |  |  |
| **Infant sex** |  |  |  |  |  |  |  |
| Male | 496 (51.1) | Reference | | | Reference | | |
| Female | 474 (48.9) | 1.14 | 0.87-1.49 | 0.350 | 1.13 | 0.86-1.48 | 0.390 |
| Missing | 0 (0.0) |  |  |  |  |  |  |
| **Plurality** |  |  |  |  |  |  |  |
| Singleton | 934 (96.3) | Reference | | | Reference | | |
| ≥2 | 36 (3.7) | 0.91 | 0.43-1.94 | 0.813 | 0.92 | 0.43-1.95 | 0.820 |
| Missing | 0 (0.0) |  |  |  |  |  |  |
| **Birth order** |  |  |  |  |  |  |  |
| 1st | 743 (76.6) | Reference | | | Reference | | |
| 2nd | 143 (14.7) | 1.29 | 0.91-1.83 | 0.157 | 1.32 | 0.93-1.88 | 0.122 |
| ≥3rd | 61 (6.3) | 0.58 | 0.28-1.18 | 0.129 | 0.54 | 0.25-1.15 | 0.108 |
| Continuous |  | 0.91 | 0.74-1.12 | 0.374 | 0.90 | 0.72-1.12 | 0.351 |
| Missing | 23 (2.4) |  |  |  |  |  |  |
| **Size for gestational age** |  |  |  |  |  |  |  |
| <10^th^ percentile | 115 (11.9) | 1.21 | 0.81-1.81 | 0.351 | 1.11 | 0.73-1.69 | 0.632 |
| 10^th^_-_90^th^ percentile | 741 (76.4) | Reference | | | Reference | | |
| >90^th^ percentile | 98 (10.1) | 0.88 | 0.55-1.41 | 0.592 | 0.91 | 0.56-1.47 | 0.691 |
| Missing | 16 (1.6) |  |  |  |  |  |  |
| **Gestational age** |  |  |  |  |  |  |  |
| <37 weeks | 127 (13.1) | 0.73 | 0.47-1.15 | 0.181 | 0.72 | 0.46-1.13 | 0.155 |
| 37-41 weeks | 803 (82.8) | Reference | | | Reference | | |
| ≥42 | 24 (2.5) | 1.42 | 0.67-3.02 | 0.365 | 1.49 | 0.69-3.21 | 0.298 |
| Continuous |  | 1.03 | 0.97-1.11 | 0.337 | 1.04 | 0.97-1.12 | 0.229 |
| Missing | 16 (1.6) |  |  |  |  |  |  |
| **Delivery type** |  |  |  |  |  |  |  |
| Vaginal spontaneous | 613 (63.2) | Reference | | | Reference | | |
| Vaginal forceps or vacuum | 81 (8.4) | 0.64 | 0.36-1.16 | 0.140 | 0.59 | 0.32-1.10 | 0.099 |
| Cesarean | 275 (28.3) | 1.05 | 0.78-1.42 | 0.741 | 1.09 | 0.79-1.47 | 0.600 |
| Missing | 1 (0.1) |  |  |  |  |  |  |
| **Birth weight (g)** |  |  |  |  |  |  |  |
| <2500 | 70 (7.2) | 0.76 | 0.42-1.36 | 0.350 | 0.66 | 0.36-1.23 | 0.190 |
| 2500-3999 | 815 (84.0) | Reference | | | Reference | | |
| ≥4000 | 85 (8.8) | 0.63 | 0.36-1.11 | 0.113 | 0.69 | 0.39-1.22 | 0.197 |
| Continuous |  | 0.99 | 0.99-1.00 | 0.954 | 1.00 | 0.99-100 | 0.457 |
| Missing |  |  |  |  |  |  |  |
| **Maternal BMI ^c^** |  |  |  |  |  |  |  |
| <18.5 | 8 (3.8) | 0.55 | 0.07-4.11 | 0.556 | 0.57 | 0.07-4.36 | 0.587 |
| 18.5-24.9 | 99 (47.6) | Reference | | | Reference | | |
| 25-29.9 | 50 (24.3 | 0.40 | 0.14-1.19 | 0.100 | 0.39 | 0.13-1.16 | 0.092 |
| ≥30 | 49 (23.8) | 0.52 | 0.19-1.40 | 0.196 | 0.51 | 0.19-1.38 | 0.186 |
| Continuous |  | 0.95 | 0.88-1.02 | 0.137 | 0.95 | 0.88-1.02 | 0.145 |
| Missing | 1 (0.5) |  |  |  |  |  |  |
| **Maternal smoking** |  |  |  |  |  |  |  |
| No | 882 (91.0) | Reference | | | Reference | | |
| Yes | 73 (7.5) | 0.99 | 0.59-1.66 | 0.998 | 1.06 | 0.63-1.79 | 0.814 |
| Missing | 15 (1.5) |  |  |  |  |  |  |

^a^ Adjusted for birth year, sex, maternal race/ethnicity, maternal education, and tumor malignancy

^b^ Bonferroni corrected reference *P values*: 0.003 for an experiment-wide significance of 0.05

^c^ Pre-pregnancy maternal body mass index (BMI) data collection began in 2005
