# Supplementary material for: Maternal and perinatal factors are associated with risk of pediatric central nervous system tumors and poorer survival after diagnosis
Source: Sci Rep. 2021 May 17;11:10410. doi: 10.1038/s41598-021-88385-3 (PMC8129132; doi:10.1038/s41598-021-88385-3)
Supplement: Supplementary file 7 — Supplementary Table 7. [file 41598_2021_88385_MOESM7_ESM.docx]

Supplementary Table 7. Non-significant associations between maternal and perinatal factors and survival of pediatric ependymoma cases

| **Maternal and perinatal characteristics** | **Ependymoma** | | | | | | |
| --- | --- | --- | --- | --- | --- | --- | --- |
|  | **Cases** | **Unadjusted model** | | | **Adjusted model ^a^** | | |
|  |  | **HR** | **95%CI** | **p-value ^b^** | **HR** | **95%CI** | **p-value ^b^** |
| **Maternal race/ethnicity** |  |  |  |  |  |  |  |
| Non-Hispanic White | 61 (38.8) | Reference | | | Reference | | |
| Non-Hispanic Black | 10 (6.4) | 0.73 | 0.17-3.18 | 0.676 | 0.71 | 0.16-3.14 | 0.657 |
| Hispanic | 82 (52.2) | 1.23 | 0.67-2.27 | 0.501 | 1.38 | 0.71-2.68 | 0.342 |
| Other | 4 (2.6) | 0.88 | 0.12-6.64 | 0.900 | 0.69 | 0.09-5.28 | 0.720 |
| Missing | 0 (0.0) |  |  |  |  |  |  |
| **Maternal age** |  |  |  |  |  |  |  |
| <25 | 69 (43.9) | 0.97 | 0.49-1.90 | 0.925 | 1.12 | 0.54-2.29 | 0.768 |
| 25-29 | 38 (24.2) | Reference | | | Reference | | |
| 30-34 | 35 (22.3) | 0.52 | 0.19-1.39 | 0.191 | 0.49 | 0.19-1.31 | 0.155 |
| ≥35 | 15 (9.6) | 1.17 | 0.42-3.29 | 0.762 | 1.00 | 0.32-3.11 | 0.994 |
| Continuous |  | 0.98 | 0.94-1.03 | 0.543 | 0.96 | 0.91-1.02 | 0.175 |
| Missing | 0 (0.0) |  |  |  |  |  |  |
| **Maternal education** |  |  |  |  |  |  |  |
| < High school | 50 (31.9) | 0.76 | 0.37-1.54 | 0.442 | 0.67 | 0.32-1.42 | 0.300 |
| High school | 59 (37.5) | Reference | | | Reference | | |
| > High school | 44 (28.0) | 1.26 | 0.64-2.51 | 0.506 | 1.39 | 0.69-2.79 | 0.361 |
| Missing | 4 (2.6) |  |  |  |  |  |  |
| **Residence on Mexican border** |  |  |  |  |  |  |  |
| No | 128 (81.5) | Reference | | | Reference | | |
| Yes | 29 (18.5) | 1.18 | 0.60-2.32 | 0.628 | 0.94 | 0.44-2.02 | 0.874 |
| Missing | 0 (0.0) |  |  |  |  |  |  |
| **Maternal residency** |  |  |  |  |  |  |  |
| Urban | 132 (84.1) | Reference | | | Reference | | |
| Rural | 6 (3.8) | 1.50 | 0.36-6.27 | 0.576 | 1.96 | 0.45-8.51 | 0.370 |
| Missing | 19 (12.1) |  |  |  |  |  |  |
| **Infant sex** |  |  |  |  |  |  |  |
| Male | 90 (57.3) | Reference | | | Reference | | |
| Female | 67 (42.7) | 1.12 | 0.63-1.98 | 0.699 | 1.11 | 0.62-1.99 | 0.729 |
| Missing | 0 (0.0) |  |  |  |  |  |  |
| **Plurality** |  |  |  |  |  |  |  |
| Singleton | 152 (96.8) | Reference | | | Reference | | |
| ≥2 | 5 (3.2) | 1.44 | 0.35-5.96 | 0.614 | 1.75 | 0.41-7.57 | 0.452 |
| Missing | 0 (0.0) |  |  |  |  |  |  |
| **Birth order** |  |  |  |  |  |  |  |
| 1st | 128 (81.5) | Reference | | | Reference | | |
| 2nd | 19 (12.1) | 1.35 | 0.60-3.02 | 0.466 | 1.53 | 0.66-3.55 | 0.323 |
| ≥3rd | 7 (4.5) | - | - | - | - | - | - |
| Continuous |  | 0.77 | 0.42-1.41 | 0.392 | 0.77 | 0.41-1.45 | 0.413 |
| Missing |  |  |  |  |  |  |  |
| **Size for gestational age** |  |  |  |  |  |  |  |
| <10^th^ percentile | 25 (15.9) | 0.63 | 0.27-1.49 | 0.291 | 0.69 | 0.29-1.66 | 0.409 |
| 10^th^_-_90^th^ percentile | 118 (75.2) | Reference | | | Reference | | |
| >90^th^ percentile | 13 (8.3) | 0.59 | 0.18-1.89 | 0.372 | 0.54 | 0.16-1.77 | 0.311 |
| Missing | 1 (0.6) |  |  |  |  |  |  |
| **Gestational age** |  |  |  |  |  |  |  |
| <37 weeks | 21 (13.4) | 0.45 | 0.14-1.45 | 0.181 | 0.31 | 0.07-1.29 | 0.107 |
| 37-41 weeks | 127 (80.9) | Reference | | | Reference | | |
| ≥42 | 8 (5.1) | 0.68 | 0.16-2.82 | 0.597 | 0.64 | 0.15-2.72 | 0.548 |
| Continuous |  | 1.02 | 0.89-1.18 | 0.732 | 1.06 | 0.91-1.23 | 0.471 |
| Missing | 1 (0.6) |  |  |  |  |  |  |
| **Delivery type** |  |  |  |  |  |  |  |
| Vaginal spontaneous | 101 (64.3) | Reference | | | Reference | | |
| Vaginal forceps or vacuum | 11 (7.0) | 1.11 | 0.39-3.16 | 0.843 | 1.17 | 0.39-3.47 | 0.773 |
| Cesarean | 45 (28.7) | 1.14 | 0.60-2.15 | 0.693 | 1.09 | 0.55-2.14 | 0.809 |
| Missing |  |  |  |  |  |  |  |
| **Birth weight (g)** |  |  |  |  |  |  |  |
| <2500 | 16 (10.2) | 0.51 | 0.16-1.64 | 0.255 | 0.35 | 0.08-1.44 | 0.145 |
| 2500-3999 | 130 (82.8) | Reference | | | Reference | | |
| ≥4000 | 11 (7.0) | 0.97 | 0.35-2.72 | 0.961 | 0.82 | 0.25-2.68 | 0.738 |
| Continuous |  | 1.00 | 0.99-1.00 | 0.539 | 1.00 | 0.99-1.00 | 0.476 |
| Missing | 0 (0.0) |  |  |  |  |  |  |
| **Maternal BMI ^c^** |  |  |  |  |  |  |  |
| <18.5 | 1 (2.6) | - | - | - | - | - | - |
| 18.5-24.9 | 25 (64.1) | Reference | | | Reference | | |
| 25-29.9 | 5 (12.8) | - | - | - | - | - | - |
| ≥30 | 8 (20.5) | 1.89 | 0.36-10.09 | 0.455 | 1.73 | 0.30-9.89 | 0.535 |
| Continuous |  | 1.04 | 0.91-1.19 | 0.540 | 1.07 | 0.93-1.23 | 0.361 |
| Missing | 0 (0.0) |  |  |  |  |  |  |
| **Maternal smoking** |  |  |  |  |  |  |  |
| No | 147 (93.6) | Reference | | | Reference | | |
| Yes | 10 (6.4) | 0.98 | 0.31-3.16 | 0.976 | 1.05 | 0.29-3.73 | 0.935 |
| Missing | 0 (0.0) |  |  |  |  |  |  |

^a^ Adjusted for birth year, sex, maternal race/ethnicity, maternal education, and tumor malignancy

^b^ Bonferroni corrected reference *P values*: 0.003 for an experiment-wide significance of 0.05

^c^ Pre-pregnancy maternal body mass index (BMI) data collection began in 2005
