# Supplementary material for: Maternal and perinatal factors are associated with risk of pediatric central nervous system tumors and poorer survival after diagnosis
Source: Sci Rep. 2021 May 17;11:10410. doi: 10.1038/s41598-021-88385-3 (PMC8129132; doi:10.1038/s41598-021-88385-3)
Supplement: Supplementary file 8 — Supplementary Table 8. [file 41598_2021_88385_MOESM8_ESM.docx]

Supplementary Table 8. Non-significant associations between maternal and perinatal factors and survival of pediatric medulloblastoma cases

| **Maternal and perinatal characteristics** | **Medulloblastoma** | | | | | | |
| --- | --- | --- | --- | --- | --- | --- | --- |
|  | **Cases** | **Unadjusted model** | | | **Adjusted model ^a^** | | |
|  |  | **HR** | **95%CI** | **p-value ^b^** | **HR** | **95%CI** | **p-value ^b^** |
| **Maternal race/ethnicity** |  |  |  |  |  |  |  |
| Non-Hispanic White | 98 (45.2) | Reference | | | Reference | | |
| Non-Hispanic Black | 17 (7.8) | 0.19 | 0.03-1.37 | 0.099 | 0.19 | 0.03-1.39 | 0.101 |
| Hispanic | 96 (44.2) | 1.55 | 0.93-2.59 | 0.090 | 1.53 | 0.87-2.69 | 0.138 |
| Other | 6 (2.8) | 1.30 | 0.31-5.50 | 0.721 | 1.26 | 0.29-5.38 | 0.754 |
| Missing | 0 (0.0) |  |  |  |  |  |  |
| **Maternal age** |  |  |  |  |  |  |  |
| <25 | 79 (36.4) | 1.16 | 0.64-2.09 | 0.628 | 1.21 | 0.64-2.31 | 0.553 |
| 25-29 | 63 (29.0) | Reference | | | Reference | | |
| 30-34 | 52 (24.0) | 0.75 | 0.36-1.54 | 0.431 | 0.81 | 0.39-1.69 | 0.576 |
| ≥35 | 23 (10.6) | 1.06 | 0.45-2.53 | 0.893 | 0.99 | 0.41-2.38 | 0.976 |
| Continuous |  | 0.99 | 0.96-1.04 | 0.843 | 0.99 | 0.96-1.04 | 0.816 |
| Missing | 0 (0.0) |  |  |  |  |  |  |
| **Maternal education** |  |  |  |  |  |  |  |
| < High school | 63 (29.0) | 1.51 | 0.79-2.88 | 0.209 | 1.27 | 0.64-2.51 | 0.491 |
| High school | 64 (29.5) | Reference | | | Reference | | |
| > High school | 87 (40.1) | 1.16 | 0.62-2.17 | 0.650 | 1.26 | 0.66-2.41 | 0.482 |
| Missing | 3 (1.4) |  |  |  |  |  |  |
| **Maternal nativity** |  |  |  |  |  |  |  |
| U.S. born | 166 (76.6) | Reference | | | Reference | | |
| Mexico | 40 (18.4) | 1.70 | 0.95-3.07 | 0.075 | 1.22 | 0.59-2.54 | 0.594 |
| Other | 10 (4.6) | 1.87 | 0.67-5.23 | 0.232 | 2.10 | 0.62-7.18 | 0.235 |
| Missing | 1 (0.4) |  |  |  |  |  |  |
| **Residence on Mexican border** |  |  |  |  |  |  |  |
| No | 188 (86.6) | Reference | | | Reference | | |
| Yes | 29 (13.4) | 1.43 | 0.76-2.68 | 0.265 | 1.01 | 0.49-2.03 | 0.985 |
| Missing | 0 (0.0) |  |  |  |  |  |  |
| **Maternal residency** |  |  |  |  |  |  |  |
| Urban | 179 (82.5) | Reference | | | Reference | | |
| Rural | 10 (4.6) | 1.40 | 0.44-4.50 | 0.569 | 1.84 | 0.55-6.20 | 0.323 |
| Missing | 28 (12.9) |  |  |  |  |  |  |
| **Infant sex** |  |  |  |  |  |  |  |
| Male | 143 (65.9) | Reference | | | Reference | | |
| Female | 74 (34.1) | 0.83 | 0.48-1.42 | 0.490 | 0.76 | 0.44-1.33 | 0.340 |
| Missing | 0 (0.0) |  |  |  |  |  |  |
| **Plurality** |  |  |  |  |  |  |  |
| Singleton | 212 (97.7) | Reference | | | Reference | | |
| ≥2 | 5 (2.3) | 2.61 | 0.82-8.36 | 0.105 | 2.97 | 0.90-9.73 | 0.073 |
| Missing | 0 (0.0) |  |  |  |  |  |  |
| **Birth order** |  |  |  |  |  |  |  |
| 1st | 162 (74.7) | Reference | | | Reference | | |
| 2nd | 43 (19.7) | 1.24 | 0.69-2.22 | 0.478 | 1.19 | 0.64-2.21 | 0.581 |
| ≥3rd | 6 (2.8) | 2.27 | 0.70-7.34 | 0.172 | 2.31 | 0.68-7.89 | 0.181 |
| Continuous |  | 1.41 | 0.94-2.11 | 0.099 | 1.39 | 0.91-2.12 | 0.123 |
| Missing | 6 (2.8) |  |  |  |  |  |  |
| **Gestational age** |  |  |  |  |  |  |  |
| <37 weeks | 30 (13.8) | 1.36 | 0.69-2.69 | 0.379 | 1.24 | 0.59-2.57 | 0.571 |
| 37-41 weeks | 179 (82.5) | Reference | | | Reference | | |
| ≥42 | 6 (2.8) | 1.13 | 0.27-4.62 | 0.870 | 1.06 | 0.25-4.51 | 0.937 |
| Continuous |  | 0.99 | 0.89-1.09 | 0.822 | 0.99 | 0.89-1.11 | 0.913 |
| Missing | 2 (0.9) |  |  |  |  |  |  |
| **Delivery type** |  |  |  |  |  |  |  |
| Vaginal spontaneous | 140 (64.5) | Reference | | | Reference | | |
| Vaginal forceps or vacuum | 11 (5.1) | 1.23 | 0.44-3.44 | 0.695 | 1.43 | 0.48-4.32 | 0.523 |
| Cesarean | 66 (30.4) | 1.03 | 0.60-1.77 | 0.902 | 1.07 | 0.62-1.85 | 0.804 |
| Missing | 0 (0.0) |  |  |  |  |  |  |
| **Birth weight (g)** |  |  |  |  |  |  |  |
| <2500 | 18 (8.3) | 1.52 | 0.69-3.37 | 0.299 | 1.41 | 0.59-3.40 | 0.439 |
| 2500-3999 | 176 (81.1) | Reference | | | Reference | | |
| ≥4000 | 23 (10.6) | 1.54 | 0.77-3.06 | 0.222 | 1.46 | 0.70-3.03 | 0.310 |
| Continuous |  | 0.99 | 0.99-1.00 | 0.719 | 0.99 | 0.99-1.00 | 0.606 |
| Missing | 0 (0.0) |  |  |  |  |  |  |
| **Maternal BMI ^c^** |  |  |  |  |  |  |  |
| <18.5 | 2 (4.9) | - | - | - | - | - | - |
| 18.5-24.9 | 26 (63.4) | Reference | | | Reference | | |
| 25-29.9 | 8 (19.5) | 1.79 | 0.54-5.96 | 0.340 | 1.57 | 0.46-5.32 | 0.467 |
| ≥30 | 5 (12.2) | - | - | - | - | - | - |
| Continuous |  | 1.00 | 0.91-1.11 | 0.950 | 1.02 | 0.91-1.14 | 0.759 |
| Missing | 0 (0.0) |  |  |  |  |  |  |
| **Maternal smoking** |  |  |  |  |  |  |  |
| No | 205 (94.5) | Reference | | | Reference | | |
| Yes | 9 (4.1) | 1.35 | 0.49-3.73 | 0.561 | 1.42 | 0.50-4.04 | 0.509 |
| Missing | 3 (1.4) |  |  |  |  |  |  |

^a^ Adjusted for birth year, sex, maternal race/ethnicity, maternal education, and tumor malignancy

^b^ Bonferroni corrected reference *P values*: 0.003 for an experiment-wide significance of 0.05

^c^ Pre-pregnancy maternal body mass index (BMI) data collection began in 2005
